# The impact of digital technology on sports consumption: evidence from Chinese college students

**DOI:** 10.3389/fpsyg.2025.1501327

**Published:** 2025-05-20

**Authors:** Yuan-Ji Zhong, Jiang-Wei Yang, Wen-Hao Guo, Yong-Shun Wang

**Affiliations:** ^1^School of Physical Education and Arts, Jiangxi University of Science and Technology, Ganzhou, China; ^2^Department of Physical Education, Xiamen Institute of Technology, Xiamen, China; ^3^College of Physical Education, Huaqiao University, Quanzhou, China

**Keywords:** digital technology use, sports consumption behavior, emotional experience, symbolic perception, college students

## Abstract

**Background:**

In an increasingly digitalized world, the impact of digital technology on sports consumption behavior is a pivotal area of study, particularly among college students who are highly engaged with digital platforms.

**Methods:**

This study investigates how digital technology use shapes college students’ sports consumption behavior, incorporating emotional experience as a mediator and symbolic perception as a moderator. Guided by the Stimulus-Organism-Response (S-O-R) framework and Symbolic Consumption Theory (SCT), data from 861 Chinese college students (511 males and 350 females) were analyzed using Structural Equation Modeling (SEM).

**Results:**

Results reveal that digital technology use significantly enhances sports consumption, not only through a direct effect but also indirectly by improving emotional experience. Furthermore, symbolic perception amplifies the effect of emotional experience on consumption behavior. These findings highlight the dual psychological pathways through which technology influences sports-related decisions.

**Conclusion:**

The study offers theoretical contributions by integrating emotional and symbolic mechanisms, and provides practical insights for sports marketers and educators aiming to engage college students in digital-era consumption.

## 1 Introduction

The global sport consumption market is witnessing remarkable and sustained expansion. As reported by Statista (2025), the market reached an estimated value of USD 387.7 billion in 2024 and is projected to grow steadily to USD 404.8 billion by 2025. Between 2023 and 2028, the compound annual growth rate (CAGR) is expected to reach 3.9%, reflecting a robust upward trend across both mature and emerging economies. This accelerating growth is influenced by multiple factors, among which the rapid proliferation and integration of digital technologies stands out as a particularly transformative force.

In an era marked by rapid digitalization, emerging technologies such as artificial intelligence, big data analytics, and advanced communication systems are deeply embedded in the fabric of everyday life. According to the *2025 Report on the Digital Transformation of Sports Entities* released by the Global Sports Innovation Center (GSIC), digital technologies are playing an increasingly vital role in the governance and operation of the sport industry. The report calls for strategic, system-wide initiatives to accelerate digital transformation as a means to enhance the sector’s overall economic competitiveness (Global Sports Innovation Center [GSIC], 2019). The deep integration of digital technologies has injected new momentum into the sports consumption market, fostering a wave of innovative formats and business models. Technological applications such as smart stadiums and unmanned fitness centers have begun to materialize, while emerging formats like virtual skiing, rowing simulations, and flight-based competitive experiences illustrate the expanding diversity of sport. These innovations not only broaden the spectrum of engagement but also fuel participation enthusiasm—particularly among younger demographics (Niu et al., 2025).

In the Chinese context, sports consumption has increasingly taken on a symbolic dimension, evolving beyond a purely material transaction into a potent vehicle for expressing identity, signaling social status, and shaping lifestyle preferences (Gordillo-Rodriguez and Sanz-Marcos, 2020). This symbolic turn is particularly salient among college students, a generation of digital natives who are not only key participants in the sports consumption market (Gentile et al., 2024), but also actively engage with sports brands, content, and experiences through various digital platforms. For this cohort, sports products and services fulfill not only functional needs but also serve as markers of personal values, social identity, and group affiliation. As such, sports consumption is progressively transforming into a form of cultural expression and social practice, reflecting the dual pursuit of self-identification and social belonging among young consumers.

This study focuses on Chinese college students as the target population and aims to investigate how digital technologies shape their sports consumption behaviors, as well as how symbolic meanings embedded in such consumption contribute to the formation of individual and social identity. By examining these interrelated dimensions, the research endeavors to elucidate the mechanisms through which digital engagement and symbolic interpretation jointly influence consumption patterns within the sports domain. The study is expected to provide both theoretical contributions and practical guidance for promoting the sustainable development of the sports consumption market in an increasingly digitalized environment.

### 1.1 S-O-R theory

The S-O-R theory, crafted by Mehrabian and Russell in 1974, extends traditional S-R theory by integrating environmental psychology perspectives (Mehrabian and Russell, 1974). It maintains the stimulus-response link and incorporates the organism’s internal mental state, which mediates the relationship. This theory is extensively applied to understand how external stimuli influence individual attitudes and behaviors, particularly in sports, where it has been used to analyze sports tourism consumption and the public’s inclination toward physical activity.

Within the S-O-R framework, stimuli are conceptualized as external environmental factors that provoke individuals’ attention, perception, cognition, or emotional responses. These stimuli may arise from a wide array of sensory, informational, or social sources that activate internal psychological processes. The organism refers to the individual’s internal system, encompassing cognitive appraisals, affective reactions, and psychological states that mediate the relationship between external stimuli and behavioral outcomes. It functions as the interpretive core, transforming external influences into subjective experiences. The response, in turn, captures the individual’s observable behavioral actions, including tendencies toward approach or avoidance, consumption decisions, or other forms of goal-directed behavior. Existing research views the emotional and cognitive responses to stimuli as part of an individual’s perception and psychological change. For instance, Luqman et al. (2017) explored how technological stress and fatigue influence users’ emotional states and decisions to discontinue Facebook use, based on the S-O-R model. Similarly, Parboteeah et al. (2009) discovered that website features impact consumers’ cognitive shopping goals and emotional experience through task-related and emotionally relevant cues, such as navigability and visual appeal.

Within the domain of sports consumption, the S-O-R theory also exhibits strong explanatory. For instance, Zhang et al. (2023) employed the S-O-R framework to examine how college students’ internet usage behavior influences their engagement with spectator sports consumption, revealing that perceived value and trust act as critical mediators in this process. Similarly, Paek et al. (2021) found that the perceived quality of sports e-commerce websites significantly impacts consumers’ shopping satisfaction and overall well-being through the mediating effect of flow experiences, further validating the applicability of the S-O-R model within digital sports commerce environments.

Building upon this theoretical foundation, the present study integrates the S-O-R framework to explore the dynamic interplay between digital technology use and sports consumption behavior, with a particular focus on uncovering the mechanisms that facilitate increased sports consumption among college students.

### 1.2 Digital technology use and sports consumption behavior

Digital technology encompasses a comprehensive spectrum of data-driven innovations, including artificial intelligence, big data analytics, cloud computing, and intelligent systems (Li et al., 2025). Its rapid advancement has fueled significant growth in the global digital economy, fostering innovation across various sectors and opening new avenues for the high-quality development of the sports consumption economy (Schmidt, 2020). Recently, academic research has increasingly focused on the intersection of digital technology and the sports economy, with particular attention to how digital advancements are reshaping sports consumption behaviors (Ruth et al., 2022). Although studies on digital technology usage predominantly explore its effects on different populations, particularly regarding behavioral and psychological health, much of the existing research has centered on cognitive development in adolescents (Haddock et al., 2022), social and psychological well-being (Sewall et al., 2020), and happiness in older adults (Wilson, 2018). However, there is a notable gap in the literature addressing how digital technology influences sports consumption behaviors among college students, highlighting the need for further investigation.

Sports consumption behavior is conceptualized as the mechanism through which individuals, recognizing and valuing the intrinsic merits of sports, address their physiological, psychological, and social needs. This process is realized via financial and credit transactions for the procurement and utilization of sports goods, services, or experiences, and is stratified into three distinct dimensions: tangible, participatory, and spectator (Horne, 2017) The tangible dimension is manifested in the acquisition of physical sporting commodities, such as athletic gear and apparel, which are fundamental for active sports engagement (Tina and Petrić, 2022). The participatory dimension is characterized by the direct consumption of sports activities and ancillary services, including recreational and fitness-oriented engagements (Lera-López and Rapún-Gárate, 2007). The spectator dimension, conversely, pertains to the patronage and appreciation of sports events and entertainments, encompassing the procurement of event tickets and the experiential aspect of sports spectatorship (Pan et al., 1997).

Digital technology captivates college students through diverse content, thereby influencing their cognitive frameworks and consumption patterns, and shaping their consumer value systems, which in turn, propels their engagement in sports consumption (Dalton and Crosby, 2013). Current academic research on sports consumption behavior is predominantly anchored within traditional consumption studies, emphasizing factors such as motivations for sports consumption (Lee and Schoenstedt, 2011), intentions to repurchase (Hui Li et al., 2024), and the dynamics between service quality and consumer satisfaction (Whitburn et al., 2020).

Although the physical exercise behaviors of college students have been extensively studied (Luo and He, 2021; Liu et al., 2023), there remains a notable gap in the literature regarding their sports consumption behaviors. In particular, few quantitative studies have systematically explored the mechanisms through which digital technology influences college students’ engagement in sports consumption. Existing evidence, though limited, has suggested a positive predictive relationship. For instance, research adopting a perceived value perspective has demonstrated that the intensity and frequency of Internet usage significantly and positively impact college students’ participation in spectator sports consumption (Zhang et al., 2023). Building upon this foundation, recent research increasingly emphasizes that Generation Z’s sports consumption behavior is deeply mediated by digital platforms, where fan culture, e-sports participation, and digital identity construction emerge as pivotal drivers. College students, representing a core subgroup of Generation Z, exhibit particularly pronounced patterns in this digitalized sports engagement. According to Deloitte (2023), nearly all Gen Z sports fans rely on social media to consume sports-related content, favoring behind-the-scenes access and direct interactions with athletes over conventional broadcast formats. The rise of e-sports has further transformed traditional modes of engagement, turning passive spectatorship into active participation through live streaming, interactive chats, and virtual competitions (Peng and Guo, 2023). These developments underscore the growing importance of experiential consumption, wherein the perceived value of sports derives not only from the events themselves but also from the immersive and interactive experiences they facilitate. In addition, digital social media enhances the dissemination of information about sports matches and teams, thereby reinforcing the loyalty of college sports fans and exerting a substantial positive influence on tangible, participatory, and spectator-based sports consumption behaviors (Clark and Maher, 2016).

Against this backdrop, the present study aims to clarify the impact mechanisms through which digital technology shapes the sports consumption behaviors of college students. Consequently, the following hypothesis (H1) is proposed:

*H1*: The Digital Technology Use has a significant direct effect on the sports consumption behavior of college students.

*H1a*: The Digital Technology Use has a significant positive effect on the tangible sports consumption behavior of college students.

*H1b*: The Digital Technology Use has a significant positive effect on the participatory sports consumption behavior of college students.

*H1c*: The Digital Technology Use has a significant positive effect on the spectator sports consumption behavior of college students.

### 1.3 Mediating effects of emotional experience

Emotion, a psychological state influenced by the evaluation of events or thoughts, includes mood and attitude (Bagozzi et al., 1999). Emotional experience is the internal reaction to external stimuli, reflecting personal emotions and attitudes (Schmitt, 1999). While research has often highlighted the negative effects of excessive digital technology use on college students’ emotions, the positive emotional experience facilitated by digital technology have been less emphasized (Cain, 2018). However, digital technology, when used appropriately, can enhance college students’ emotional experience, as seen in their use of social media for self-presentation (Yang and Bradford Brown, 2016) and the enhancement of self-identity through virtual interactions (Hefner et al., 2007). Building on this foundation, the present study conceptualizes emotional experience as predominantly reflecting positive emotions. Specifically, it encompasses feelings such as pleasure, satisfaction, and excitement, which are typically evoked through digital engagement in environments associated with sports consumption. This study thus proposes (H2):

*H2*: The Digital Technology Use has a significant positive effect on emotional experience.

The emotional experience of individuals significantly influences not just tangible product consumption but also participatory and spectator sports consumption. As leisure sports like frisbee and cycling gain popularity, consumers prioritize emotional experience in the consumption process (Zhang, 2008). Online virtual sports games have been shown to positively influence college students’ emotions, thereby enhancing their spectator sports consumption behavior (Wann et al., 2021), with emotional experience emerging as a key factor in sports consumption behavior (Zhang et al., 2012).

The S-O-R theory delineates a process where external stimuli elicit cognitive and emotional shifts within individuals, subsequently influencing their behavioral responses (Huang, 2023). Emotions, as internal motivators, are recognized for their capacity to sway goal-directed actions, with positive emotional states being conducive to consumer decision-making (Kwortnik and Ross, 2007; Bartsch and Viehoff, 2010). Digital technology, with its interactive platforms and personalized recommendations, amplifies this emotional experience, thus augmenting consumer engagement and purchase intentions (Lin et al., 2011; Funk et al., 2022).

The mediating role of emotional experience in the context of digital technology and sports consumption behavior is twofold: it arises from the enhanced information acquisition facilitated by digital technology, which leads to more robust emotional reactions, and from the instant feedback and social interaction provided by virtual platforms, which bolster user participation and satisfaction (Lin et al., 2011; Funk et al., 2022). Theoretical and empirical insights suggest that emotional experience, derived from digital technology use, significantly influence sports consumption behaviors among college students. Based on this premise, the study advances the following hypotheses:

*H3*: Emotional experience has a significant positive effect on college students’ sports consumption behavior.

*H4*: Emotional experience plays a mediating role between digital technology use on college students’ sports consumption behavior.

### 1.4 The moderating role of symbolic perception

Symbolic perception is defined as an individual’s capacity to recognize and interpret the symbolic value embedded in sports consumption. It reflects how consumers perceive products, brands, and participation experiences not merely as functional entities, but as representations of personal identity, social image, and lifestyle affiliation (Zhao and Wang, 2023). In sports consumption, symbolic perception plays a multifaceted moderating role, influencing not only consumer purchasing decisions but also their emotional experience and social interactions. SCT posits that consumer behavior goes beyond material satisfaction to express identity, individuality, lifestyle, and social status (Baudrillard, 1998). Sports consumers seek social recognition and personal image through their purchases, viewing them as symbolic capital.

The Self-concept Theory, as articulated by Sirgy (1982), reveals that consumers select products or services aligning with their self-image for self-expression and social recognition. This is particularly evident in sports, where consumers purchase licensed merchandise to demonstrate loyalty, identity, and a desire for social group recognition (Hee Kwak and Kang, 2009). Social Identity Theory builds on this, highlighting the role of group identification in sports consumption behavior (Kural and Özbek, 2023).

Integrating these theories provides a nuanced view of symbolic perception’s moderating effect. It enhances the relationship between emotional experience and sports consumption by reinforcing self-consistency and group identity. Research indicates that sports consumption can enhance individual well-being through perceived social support and self-esteem, which in turn shapes the emotional experience of consumers (Guo et al., 2023). In the context of college students, symbolic perception amplifies the emotional experience associated with sports activities or products, influencing their consumption behavior (Foroudi et al., 2018; Zhao and Wang, 2023). Therefore, the study proposes hypotheses (H5):

*H5*: Symbolic perception moderates the relationship between emotional experience and sports consumption behavior.

**FIGURE 1:**
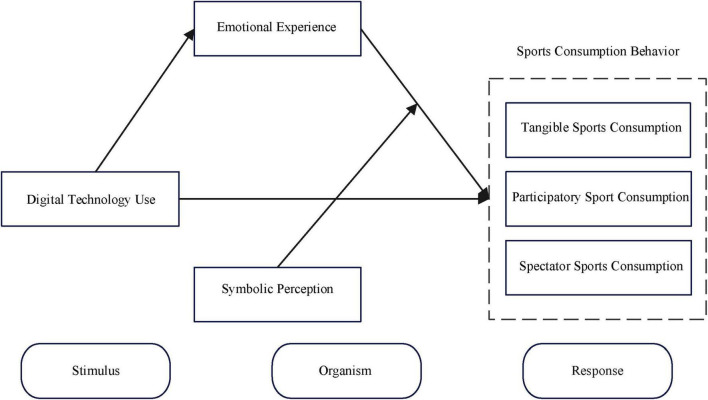
Theoretical model framework.

Based on the synthesis of the above hypotheses and reference to existing research results, the hypothesis model of this paper is established, as shown in Figure 1.

## 2 Materials and methods

Although Institutional Review Board (IRB) approval is not mandated in China, we adhered to standard IRB procedures. For instance, no personal identification information was collected. Additionally, a cover letter detailing the study’s purpose and emphasizing the voluntary and confidential nature of participation was provided to all participants. The study protocol was approved by the ethical committee of Jiangxi University of Science and Technology.

### 2.1 Research design

This study targeted college students aged 18–25 who participate in sports at least twice weekly. To achieve a diverse and representative sample, the survey was administered both online using Questionnaire Star and offline across various regions in China. Participants were informed about the study’s objectives, provided with detailed instructions for questionnaire completion, and assured of the confidentiality of their responses. The college students completed the questionnaires anonymously.

This study employed a cross-sectional research design to investigate the impact of digital technology use on sports consumption behavior among college students, with particular emphasis on the mediating role of emotional experience and the moderating role of symbolic perception. The sample was drawn from college students across six regions in China, namely Beijing, Shanghai, Wuhan, Nanchang, Chongqing, and Xi’an, ensuring a broad representation of both urban and rural populations.

To enhance the representativeness of the sample, a combination of stratified sampling and convenience sampling was employed. Specifically, stratified sampling was first used to ensure proportional representation across key demographic strata of the college student population, including geographic region (Eastern, Central, and Western China), university type (comprehensive vs. applied universities), and academic year (freshman to senior). Within each stratum, participants were then selected using convenience sampling based on accessibility and voluntary participation.

The inclusion criteria were as follows: participants (1) were between 18 and 25 years old, (2) engaged in sports activities at least twice per week, and (3) voluntarily consented to participate after receiving full disclosure of the study’s objectives and data confidentiality measures. Respondents with incomplete or logically inconsistent answers were excluded from the final dataset.

To uphold the quality and interpretability of the dataset, a rigorous exclusion protocol was adopted to minimize the influence of inattentive or non-deliberative responses. This protocol focused on identifying response patterns that deviated from expected cognitive engagement, thereby safeguarding the reliability of subsequent analyses. One exclusion criterion concerned completion time. Responses submitted in under 60 s were removed from the sample, as such rapid completions were considered incompatible with the cognitive processing required to thoughtfully answer the questionnaire items. The threshold was determined based on pre-survey observations and the average time needed for engaged participation. Another exclusion condition targeted monotonic response behavior. Specifically, entries in which 80% or more of the items shared identical response values were flagged as non-substantive. This uniformity was interpreted as indicative of automated or disengaged answering, likely reflecting superficial participation rather than considered judgment.

### 2.2 Measures

The questionnaire was meticulously developed through a thorough review of relevant literature and by leveraging established scales and research constructs from esteemed scholars. The study’s core variables encompass digital technology use, emotional experience, symbolic perception, and sports consumption behavior. Specifically, sports consumption behavior is the dependent variable, digital technology use is the independent variable, emotional experience serves as the mediating variable, and symbolic perception is the moderating variable. A Likert 5-point scale was employed, with responses ranging from “not at all consistent” to “completely consistent.”

#### 2.2.1 Digital technology use

The variable Digital Technology Use (DTU). Drawing from the work of Bai et al. (2023), digital technology use was assessed with three items, categorized into dimensions such as information query, online consumption, and general usage. Examples include DTU1: “You frequently use the Internet (including cell phones);” DTU2: “You frequently use the Internet to search for sports information;” and DTU3: “You frequently engage in online consumption.” Responses to these items were recorded on a 5-point scale to quantify the extent of digital technology use. The questionnaire i reliability in our study was evident, with a Cronbachhno α value of 0.806, indicating high internal consistency.

#### 2.2.2 Emotional experience

The variable Emotional Experience (EE). This variable refers to the college students’ own emotional states resulting from digital technology use, was measured with three items informed by the research of Happ et al. (2021) and Yu et al. (2023), and tailored to this study’s objectives. Items include EE1: “Engaging in digital sports consumption puts me in a good mood;” EE 2: “Engaging in digital sports consumption makes me feel very satisfied;” EE3: “Engaging in digital sports consumption makes me feel very excited and thrilled.” These items are rated on a 5-point scale, where higher scores indicate more positive bond about emotional experience. In our study, the emotional experience scale exhibited strong internal consistency, as evidenced by a Cronbach’s α value of 0.805.

#### 2.2.3 Symbolic perception

The variable Symbolic Perception (SP). It understood as the evaluative perception of consumption behavior, was measured with three items developed in line with Zhao and Wang (2023) research and the study’s requirements. Items include SP1: “Engaging in high-grade sports consumption reflects my social status;” SP2: “Purchasing distinctive sports products can express my personality;” SP3: “Participating in high-quality sports consumption allows me to experience a high quality of life.” These items were rated on a 5-point scale, with higher scores indicating more effective symbolic perception. In our research, the symbolic perception scale demonstrated sound internal consistency, as reflected by Cronbach’s α value of 0.834.

#### 2.2.4 Sports consumption behavior

The variable Sports Consumption Behavior (SCB). The variable was operationalized by integrating the dimensions explored by Sun et al. (2001) and others, consolidating “Rehabilitation-Type Consumption,” “Game-Type Consumption,” and “Activity-Type Consumption” into “Participatory Sports Consumption.” The construct was ultimately measured by three dimensions: “Tangible Sports Consumption” (TSC), “Participatory Sports Consumption” (PSC), and “Spectator Sports Consumption” (SSC), with items such as TSC1: “Purchasing sports equipment and facilities,” SSC2: “Purchasing tickets for sports performances,” and PSC3: “Purchasing paid fitness tickets,” among nine other items. These items were rated on a 5-point scale. A higher tally on this scale suggests a more frequent occurrence of sports consumption behaviors. The Cronbach hi α value for this specific scale in our research was 0.865, indicating a satisfactory level of internal consistency.

### 2.3 Data collection

Employing G*Power 3.1.9.7 software, the minimum sample size required was calculated to be 146 participants, considering an effect size (f^2^) of 0.15, a significance level (α) of 0.05, a statistical power (1-β) of 0.95, and the inclusion of six predictor variables. The survey was conducted from March to May 2024 in Beijing, Shanghai, Wuhan, Nanchang, Chongqing, and Xi’an, encompassing both a pre-survey and the formal survey phase. In the pre-survey phase in March, 100 questionnaires were distributed across Shanghai, Nanchang, and Chongqing, yielding 92 valid responses, which corresponds to a recovery rate of 93.47%. The pre-survey data underwent reliability and validity testing, focusing on constructs such as digital technology use, emotional experience, symbolic perception, and various dimensions of sports consumption.

Data were collected using a combination of online and offline survey methods, facilitated through the Questionnaire Star platform for digital distribution and in-person questionnaires for offline collection. This hybrid approach enhanced both the accessibility and reliability of the collected data. Data collection took place between March and May 2024, initially recruiting 900 students. After data cleaning, which involved the exclusion of 39 incomplete responses, a final sample of 861 valid responses was obtained, yielding a participation rate of 95.66%.

### 2.4 Data analysis

All statistical analyses were conducted using IBM SPSS Statistics 26 (IBM Corporation, Armonk, NY, United States) and Amos 26 (IBM Corporation, Armonk, NY, United States). The analysis proceeded in three stages to ensure a comprehensive and rigorous examination of the relationships among the variables.

First, descriptive statistics were calculated to summarize participants demographic characteristics. To assess potential common method bias, the Harman single-factor test and the Unmeasured Latent Method Covariate (ULMC) approach were conducted, with results indicating that common method variance was not a serious concern. Reliability analysis was performed to assess the internal consistency of the scales used to measure each variable, with Cronbachhe scales used hat common ingipto confirm that all scales achieved acceptable reliability. Pearson correlation analysis was used to examine the relationships between key variables.

Second, SEM was employed using Amos 26. Confirmatory factor analysis (CFA) was conducted to evaluate the construct validity of the measurement model, ensuring that the latent variables were accurately represented by their indicators. Model fit was assessed using multiple fit indices to confirm the adequacy of the hypothesized model. Path analysis was then performed to evaluate the standardized coefficients for each hypothesized relationship.

Third, to assess mediating and moderating effects, mediation analysis was conducted using AMOS 26.0, with bootstrapping procedures based on 1,000 resamples. Both bias-corrected and percentile-based 95% confidence intervals were generated to test the significance of indirect effects. Additionally, the moderation effect of symbolic perception on the relationship between emotional experience and sports consumption behavior was tested using the PROCESS macro for SPSS 26.0 through interaction terms. Simple slope analysis was further performed to explore the nature and directionality of the moderating effect.

## 3 Results

### 3.1 Common method bias test

This study employed the Harman single factor test and the ULMC test to assess common method bias. The Harman single-factor test, conducted in SPSS 26.0, revealed that the first unrotated factor accounted for a variance of 38.714%, which is below the threshold of 40%. This finding suggests that common method bias is likely within an acceptable range.

Furthermore, the ULMC test was conducted in AMOS 26.0 by introducing an unmeasured common method factor as a latent variable within the structural equation model. This modification was made to observe changes in model fit upon the inclusion of this latent method factor. All indicators were correlated with the common method factor in a two-factor model that included both the common method factor and the distinct factors for digital technology use, emotional experience, symbolic perception, and sports consumption behavior. This model was compared with a model containing only the distinct factors. The results indicated no significant difference between the two models: Δχ^2^/Δdf = 1.163, *p* = 0.292 > 0.05, ΔNFI = 0.002, ΔTLI = 0.000, ΔRMSEA = 0.014. These findings further confirm the absence of significant common method bias in the study.

### 3.2 Descriptive statistical analysis

The descriptive statistical analysis utilizing SPSS disclosed a well-balanced academic distribution among the survey participants, with freshmen and seniors representing 25.9 and 26.8% of the sample, respectively. Monthly expenditure among participants was most frequently within the 1,500–2,500-yuan range, followed by the 800–1,500-yuan bracket. The least common expenditure categories were less than 800 yuan and over 2,500-yuan, accounting for 11 and 9% of respondents, respectively.

In relation to digital engagement with sports information, 58.1% of students reported accessing online sports content 4–7 times per week, and 59.2% spent 1–2 h per week on these activities. Financially, 32.1% of students allocated a monthly expenditure of 150–200 yuan to sports, while 22% spent more than 200 yuan monthly. The primary modes of sports consumption were identified as Tangible and participatory, with spectator sports consumption being less prevalent (Table 1).

**TABLE 1:** Sample demographic characteristics.

Item	Variable	Percentage (%)	Item	Variable	Percentage (%)
Gender	Male	59.3	Frequency of weekly digital sports information engagement	0–1	8
	Female	40.7		2–3	12.9
Grade	Freshman	25.9		4–5	27.1
	Sophomore	22.8		6–7	31
	Junior	24.5		>8	21
	Senior	26.8	Time of weekly digital sports information engagement	<0.5	11
Origin	City	51.6		0.5–1	13
	Village	48.4		1–1.5	26
Average monthly consumption level (yuan)	<800	11		1.5–2	33.2
	800–1,500	26.8		>2	16.8
	1,500–2,000	34	Sport consumption monthly (yuan)	<50	5.6
	2,000–2,500	19		50–100	12.3
	>2500	9.2		100–150	28
				150–200	32.1
				>200	22

### 3.3 Factor analysis and reliability and validity test

The appropriateness of the data for factor analysis was ascertained through the Kaiser-Meyer-Olkin (KMO) measure and Bartlett’s test of sphericity. The KMO value was 0.902, and Bartlett’s test indicated significance at *p* < 0.05, confirming the data’s suitability for factor analysis. Exploratory factor analysis (EFA) was performed using principal component analysis, retaining factors with eigenvalues exceeding 1. Items with loadings below 0.4 were eliminated, resulting in the identification of six dimensions that accounted for 74.445% of the variance. The Cronbach 74.445 s with loadings below 0.4 were eliminated, from 0.781 to 0.860, suggesting satisfactory internal consistency.

Subsequently, CFA was conducted to validate the identified dimensions, examining the relationships among the core and latent variables (Hair et al., 1998). The composite reliability (CR) for each dimension was above the threshold of 0.7, and the average variance extracted (AVE) for each dimension surpassed 0.5, substantiating the measurement model’s convergent validity (details presented in Table 2).

**TABLE 2:** Reliability and validity test of the scale.

Latent variables	Measurement index	Factor loading	Cronbach’s α	CR	AVE
Digital Technology use (DTU)	DTU1	0.798	0.806	0.828	0.616
	DTU2	0.775			
	DTU3	0.781			
Emotional experience (EE)	EE1	0.736	0.805	0.824	0.610
	EE2	0.785			
	EE3	0.820			
Symbolic perception (SP)	SP1	0.829	0.834	0.853	0.660
	SP2	0.818			
	SP3	0.790			
Tangible sports consumption (TSC)	TSC1	0.795	0.839	0.839	0.635
	TSC2	0.812			
	TSC3	0.783			
Participatory sports consumption (PSC)	PSC1	0.815	0.860	0.863	0.613
	PSC2	0.786			
	PSC3	0.811			
	PSC4	0.715			
Spectator sports consumption (SSC)	SSC1	0.835	0.781	0.828	0.706
	SSC2	0.846			

The discriminant validity of the measurement model was established as the square root of the Average Variance Extracted (AVE) for each latent variable was found to exceed the correlation coefficients between that variable and all other latent variables (Table 3). This criterion is crucial for demonstrating that the constructs are distinct from one another, thereby reinforcing the reliability of the measurement model.

**TABLE 3:** The discriminatory validity test of potential variables.

Latent variables	SSC	PSC	TSC	SP	EE	DTU
SSC	**0.840**	–	–	–	–	–
PSC	0.519**	**0.783**	–	–	–	–
TSC	0.498**	0.572**	**0.797**	–	–	–
SP	0.412**	0.494**	0.502**	**0.812**	–	–
EE	0.468**	0.484**	0.535**	0.538**	**0.781**	–
DTU	0.504**	0.525**	0.477**	0.551**	0.585**	**0.785**

DTU, Digital Technology Use; EE, Emotional Experience; SP, Symbolic Perception; TSC, Tangible Sports Consumption; SSC, Spectator Sports Consumption; PSC, Participatory Sports Consumption. Diagonal elements in bold are the square roots of AVE;

**p < 0.01.

**FIGURE 2:**
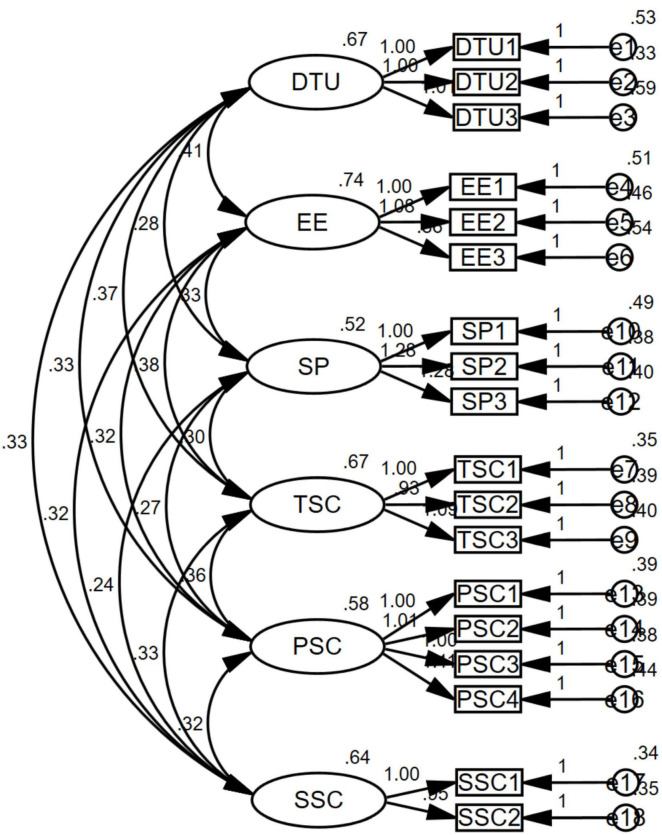
Standardized measurement model results. DTU, Digital Technology Use; EE, Emotional Experience; SP, Symbolic Perception; TSC, Tangible Sports Consumption; SSC, Spectator Sports Consumption; PSC, Participatory Sports Consumption.

CFA was conducted using AMOS 26.0 (Figure 2). Model fit assesses the degree of congruence between the hypothesized conceptual model and the empirical data derived from the sample (Hooper et al., 2008). In this study, model fit was evaluated using a comprehensive set of statistical indices, yielding the following results: χ^2^/df = 1.664, SRMR = 0.025, RMSEA = 0.028, GFI = 0.975, AGFI = 0.964, IFI = 0.989, CFI = 0.989, and TLI = 0.986. These values are within the acceptable thresholds, indicating a strong alignment between the theoretical constructs and the observed data (Table 4). The collective satisfaction of these metrics affirms the model’s reliability, validity, and overall fit, deeming the measurement model suitable for subsequent hypothesis testing.

**TABLE 4:** Fit indices of measurement and structural model.

Fitting index	χ^2^/df	SRMR	RMSEA	GFI	AGFI	IFI	CFI	TLI
Reference standard	<3.000	<0.080	<0.080	>0.900	>0.900	>0.900	>0.900	>0.900
Model value	1.664	0.025	0.028	0.975	0.964	0.989	0.989	0.986

### 3.4 Hypothesis testing

The path analysis, conducted in line with the SEM approach by Chin (2010), substantiated all the hypothesized relationships (Table 5). Digital technology use was found to be a significant predictor of increased tangible sports consumption, with a substantial effect size (β = 0.398, *p* < 0.001), thereby supporting hypothesis H1a. It positively influenced participatory sports consumption as well (β = 0.381, *p* < 0.001), validating hypothesis H1b, and had a similar positive impact on spectator sports consumption (β = 0.382, *p* < 0.001), confirming hypothesis H1c.

**TABLE 5:** The path coefficients of the initial structural model and hypothesis testing results.

Hypotheses	Paths	*B*	SE	*Z*-value	*P*-value	β	Result
H1a	DTU→TSC	0.398	0.050	7.970	***	0.392	Support
H1b	DTU→PSC	0.381	0.047	8.036	***	0.402	Support
H1c	DTU→SSC	0.382	0.053	7.165	***	0.383	Support
H2	DTU→EE	0.614	0.048	12.893	***	0.584	Support
H3a	EE→TSC	0.324	0.047	6.902	***	0.335	Support
H3b	EE→PSC	0.252	0.044	5.760	***	0.280	Support
H3c	EE→SSC	0.259	0.050	5.221	***	0.273	Support

DTU, Digital Technology Use; EE, Emotional Experience; SP, Symbolic Perception; TSC, Tangible Sports Consumption; SSC, Spectator Sports Consumption; PSC, Participatory Sports Consumption.

***p < 0.001.

Moreover, a significant positive association was identified between digital technology use and emotional experience (β = 0.614, *p* < 0.001), which supports hypothesis H2. Emotional experience was also found to be a significant predictor of tangible sports consumption (β = 0.324, *p* < 0.001), supporting hypothesis H3a. It positively affected participatory sports consumption (β = 0.252, *p* < 0.001), validating hypothesis H3b, and had a notable positive influence on spectator sports consumption (β = 0.259, *p* < 0.001), confirming hypothesis H3c.

### 3.5 Testing for the mediation effect

This study employed a Bootstrap procedure based on SEM using AMOS 26.0 to examine the mediating role of emotional experience between digital technology use and sports consumption behavior. A resampling procedure with 1,000 iterations was utilized, and both bias-corrected and percentile-based 95% confidence intervals were calculated to evaluate the significance of the mediation effect, determined by whether the intervals excluded zero.

As shown in Table 6, digital technology use exerts a significant positive direct effect on sports consumption behavior [β = 0.375, 95% CI (0.298, 0.462)]. Emotional experience was also found to significantly mediate this relationship, with an indirect effect estimate of 0.176 [95% CI (0.131, 0.232)]. The total effect of digital technology use on sports consumption behavior was 0.551, indicating a substantial combined influence [95% CI (0.469, 0.645)]. Importantly, neither the bias-corrected nor the percentile 95% confidence intervals for the indirect or direct effects contained zero, confirming the presence of both direct and indirect pathways.

**TABLE 6:** The mediating effects of emotional experience.

Paths	Effects	Estimate	Bootstrapping (1,000)				Ratio of effect (%)
			**Bias-corrected 95% CI**		**Percentile 95% CI**		
			**Lower**	**Upper**	**Lower**	**Upper**	
DTU→EE→SCB	Indirect effect	0.176	0.131	0.232	0.127	0.229	31.94
	Direct effect	0.375	0.298	0.462	0.299	0.462	68.05
	Total effect	0.551	0.469	0.645	0.464	0.642	

DTU, Digital Technology Use; EE, Emotional Experience; SCB, Sports Consumption Behavior.

These results suggest that digital technology use not only directly predicts sports consumption behavior but also indirectly influences it by enhancing positive emotional experiences, thereby providing empirical support for Hypothesis H4.

### 3.6 Test of the moderated mediation model

Building upon the mediation effect analysis, this study examines the potential variability in the relationship between college students’ emotional experience and sports consumption behavior, contingent upon the individual’s symbolic perception. The study evaluates and tests the moderating effect of symbolic perception to further elucidate the internal mechanism through which digital technology use influences sports consumption behavior, mediated by emotional experience.

**FIGURE 3:**
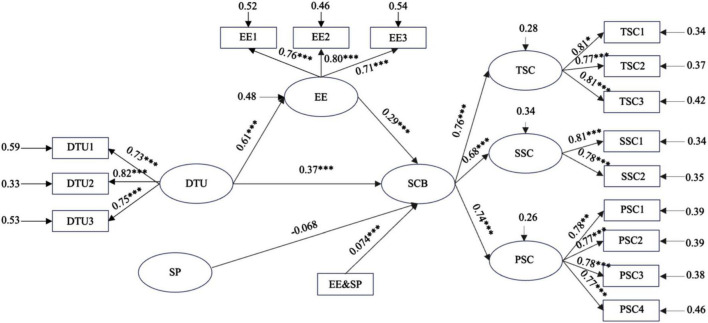
Hypothetical model. DTU, Digital Technology Use; EE, Emotional Experience; SCB, Sports Consumption Behavior; SP, Symbolic Perception; TSC, Tangible Sports Consumption; SSC, Spectator Sports Consumption; PSC, Participatory Sports Consumption; *p < 0.05; **p < 0.01; ***p < 0.001.

Utilizing Hayes’s (2013) Process v4.2, the moderating effect was tested, as depicted in Figure 3. The interaction term between emotional experience and symbolic perception was found to significantly predict sports consumption behavior (β = 0.074, *t* = 3.947, *p* < 0.001). This finding indicates that symbolic perception positively moderates the influence of emotional experience on sports consumption behavior, thereby confirming Hypothesis H5.

**FIGURE 4:**
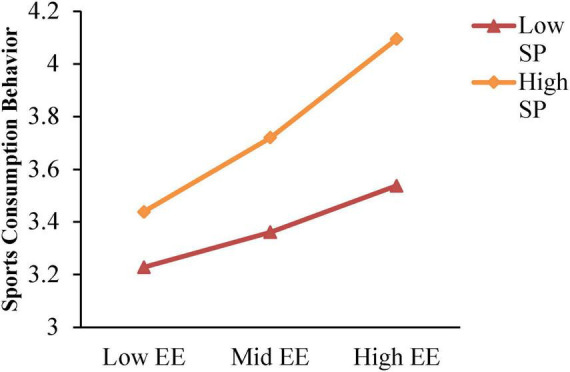
The moderating effect of symbolic perception on the impact of emotional experience on sports consumption behavior. EE, Emotional Experience; SP, Symbolic Perception.

The analysis has identified a significant predictive effect of the interaction between emotional experience and symbolic perception on college students’ sports consumption behavior. To delve deeper into the nature of this interaction, a simple slope analysis was conducted to further test the moderating effect of symbolic perception. The high symbolic perception group was defined by adding one standard deviation to the mean, while the low symbolic perception group was defined by subtracting one standard deviation from the mean. The simple slope test results, as illustrated in Figure 4, demonstrate that college students with higher levels of symbolic perception exhibit greater sports consumption behavior compared to those with lower levels, suggesting that symbolic perception has a promotional role.

The moderated mediation effect was scrutinized using the Bootstrap method, with findings detailed in Table 7. For students with lower levels of symbolic perception, emotional experience significantly and positively predicts sports consumption behavior [β = 0.065, SE = 0.017, *p* < 0.001, 95% CI (0.032, 0.098)]. Conversely, for students with higher levels of symbolic perception, the positive predictive effect of emotional experience on sports consumption behavior remains significant [β = 0.137, SE = 0.018, *p* < 0.001, 95% CI (0.101, 0.173)]. This indicates that higher levels of emotional experience are associated with increased sports consumption behavior among college students.

**TABLE 7:** The moderating role of symbolic perception.

Symbolic perception	Boot indirect effect (β)	BootSE	BootLLCI	BootULCI
M - 1 SD	0.065	0.017	0.032	0.098
M	0.101	0.013	0.076	0.127
M + 1 SD	0.137	0.018	0.101	0.173

M, Mean; SD, Standard Deviation; SE, Standard Error; BootLLCI, Bootstrapped Lower-Level Confidence Interval; BootULCI, Bootstrapped Upper-Level Confidence Interval.

## 4 Discussion

The findings of this study illuminate the intricate dynamics between digital technology use and the sports consumption behavior of college students. The research reveals the underlying complexities and intrinsic relationships at play, particularly highlighting the mediating function of emotional experience and the moderating influence of symbolic perception. Furthermore, the measurement and structural models exhibited excellent fit, with χ^2^/df = 1.664, SRMR = 0.025, RMSEA = 0.028, GFI = 0.975, AGFI = 0.964, IFI = 0.989, CFI = 0.989, and TLI = 0.986, all meeting or exceeding commonly accepted thresholds. These results confirm the robustness, reliability, and overall validity of the proposed model.

Additionally, the study engaged a convenience sample comprising 861 Chinese college students. The gender distribution within the sample was predominantly male, accounting for 59.3%, which aligns with the demographic trends observed in previous studies conducted in China (Liu et al., 2018; Zhang et al., 2018; Yang and Bradford Brown, 2016). Female participants constituted 40.7% of the sample. It is noteworthy that all participants were enrolled as full-time students, reflecting the Chinese education system’s standard practice, which predominantly supports full-time student status.

### 4.1 Digital technology use and sports consumption behavior

This study reveals a significant positive relationship between digital technology use and college students’ sports consumption behavior, supporting Hypothesis 1. The digital technology use was found to positively influence tangible, participatory, and spectator sports consumption behaviors. This outcome corroborates the notion that digital technology plays a pivotal role in fostering sports consumption, echoing Schmidt’s (2020) assertion that digital technology is a pivotal force in global development with profound implications for sports consumption patterns.

The path coefficients highlight that the influence of digital technology on tangible sports consumption (β = 0.398, *p* < 0.001) is notably strong among college students. This finding is consistent with (Hsiu-Yueh and Fei-Chuan, 2020), who emphasized that digital platforms facilitate the purchase of sports goods, thus stimulating the growth of tangible sports consumption. Additionally, the study observes a rise in the proportion of participatory (β = 0.381, *p* < 0.001) and spectator sports consumption (β = 0.382, *p* < 0.001), aligning with the work of Liu et al. (2023) and Zhang et al. (2023). These findings suggest that digital technology, through online platforms, AI, and virtual reality, offers college students a broader array of sports engagement and viewing opportunities.

Theoretically, the study’s results are explicable through the Technology Acceptance Model (TAM) and the Theory of Planned Behavior (TPB). TAM posits that usability and utility are central to technology acceptance and adoption (Davis, 1989). In this study’s context, the usability of digital technology likely encourages college students to engage in sports consumption via online platforms. TPB, on the other hand, suggests that behavioral intentions are shaped by attitudes, subjective norms, and perceived behavioral control (Ajzen, 1991). The widespread adoption of digital technology may enhance students’ attitudes toward sports consumption, with social norms and perceived convenience further propelling their engagement in sports consumption activities.

### 4.2 The mediating role of emotional experience

This study meticulously examined the mediating role of emotional experience in the relationship between digital technology use and college students’ sports consumption behavior, as posited by Hypotheses 2, 3, and 4. SEM analysis revealed that digital technology use both directly facilitates sports consumption [β = 0.375, 95% CI (0.298, 0.462)] and indirectly [β = 0.176, 95% CI (0.131, 0.232)] enhances it through the improvement of emotional experience. The use of digital technology was found to positively predict emotional experience, which subsequently significantly and positively influences tangible, participatory, and spectator sports consumption behaviors. This is in line with the Affective Transfer Theory (ATT), suggesting that emotional states can transfer across various contexts and behaviors (Park and Yun, 2024). In sports consumption, technologies like virtual reality and social media significantly impact users’ emotional experiences, with immersive and interactive features evoking strong emotions such as excitement and satisfaction, which are then channeled into sports consumption behaviors through internal affective transfer.

The study’s conclusions are corroborated by Wann et al.’s (2021) research, which identified a positive relationship between virtual networks and college students’ spectator sports consumption mediated by emotional experience. It is also important to recognize that, alongside positive emotional experiences, negative emotional experiences might also stimulate sports consumption behavior, although they may not be as widely acknowledged. Studies by Liang et al. (2024) and Zheng et al. (2020) indicate that negative emotional experiences, including impulsive, indulgent, and compulsive consumption behaviors, can similarly drive sports consumption. This highlights the pivotal role of emotional experience in the mechanism influencing sports consumption behavior within the sports domain. Thus, emotional experience emerges as a significant mediator connecting digital technology use with college students’ sports consumption behavior.

### 4.3 The moderating effect of symbolic perception

This study delved into the moderating influence of symbolic perception on the relationship between emotional experience and college students’ sports consumption behavior (β = 0.074, *t* = 3.947, *p* < 0.001). The literature and data analysis suggest that the positive effect of emotional experience on sports consumption behavior is amplified among college students with higher levels of symbolic perception. This finding is in line with self-concept theory, which suggests that individuals enhance their self-image and social status through consumption activities (Sirgy, 1982). In sports, students who identify as athletes or enthusiasts are likely to engage more in sports-related consumption behaviors when experiencing positive emotions.

Symbolic perception acts as a positive facilitator, linking emotional experience to sports consumption behavior. It allows consumers to express their identity and lifestyle through consumption, thus strengthening their emotional connection with brands (Tangsupwattana and Liu, 2018). The study’s results support Belk’s (1988) view that consumers use products to construct and express their identities. In sports, college students’ consumption of sports-related products and services serves not only physical needs but also social and psychological roles, enhancing identity value and group belonging.

This study also aligns with Richins (1994) moderated mediation model and Dubois et al. (2005) findings, which emphasize the role of digital technology in enhancing emotional experiences regulated by symbolic perception, affecting sports consumption behavior. The integration of technology in sports, such as intelligent fitness equipment and online sports communities, deepens college students’ participation and emotional and symbolic experiences.

By integrating SCT and Self-Concept Theory, this study fills a gap in the literature and offers practical guidance for leveraging digital technology to enhance college students’ sports consumption.

### 4.4 Cross-cultural variations in digital sports consumption mechanisms

While this study offers valuable insights into the mechanisms linking digital technology use, emotional experience, symbolic perception, and sports consumption behavior among Chinese college students, it is important to recognize that cultural factors may underlie these findings. The sample was drawn exclusively from China, a society distinguished by strong collectivist values, a high-context communication style, and a pronounced emphasis on conformity and group affiliation (Mattison Thompson and Brouthers, 2021).

Emerging research highlights that cultural context significantly shapes individuals’ emotional responses to digital engagement, consumption behavior, and the interpretation of symbolic value. Rabbanee et al. (2023) observed that in collectivist societies such as China, digital consumption behaviors are primarily driven by social identity motives and the pursuit of group recognition. In contrast, personal gratification and self-expression are more salient drivers in Western individualistic cultures, where digital engagement often serves to reinforce individuality rather than group belonging. Because symbolic consumption functions as a vehicle for social affiliation and identity construction, the effects observed in the Chinese sample, especially the mediating role of emotional experience and the moderating role of symbolic perception, may emerge differently in Western individualistic societies. In such contexts, digital technology may amplify individualized emotional gratifications, such as excitement and satisfaction, rather than collective symbolic alignment, as consumers seek to affirm personal uniqueness over communal ties.

Recognizing these cultural distinctions, future research would benefit from cross-cultural comparative investigations to examine the extent to which the proposed model holds across diverse cultural settings. Such inquiries could not only test the generalizability of the current findings but also reveal culturally contingent variations in the emotional, cognitive, and symbolic mechanisms that shape digital sports consumption behaviors.

### 4.5 Limitations and contributions

This study acknowledges several limitations that merit attention. Firstly, the use of convenience sampling has resulted in a sample confined to specific universities, potentially limiting the representativeness of the findings for the broader college student population across diverse geographical and cultural contexts. Future research should consider a more extensive array of institutions to ascertain the generalizability of the study’s outcomes. Secondly, reliance on self-reported data could introduce biases such as social desirability or recall bias, potentially impacting the accuracy of the results. Future studies are encouraged to employ a variety of data collection methods to bolster the reliability of their findings. Lastly, the cross-sectional design precludes the determination of causality between digital technology use and sports consumption behavior. Longitudinal research designs are recommended for future work to uncover the dynamics and long-term effects of digital technology on sports consumption patterns.

Despite these limitations, the study makes significant contributions to the field of sports consumption behavior research. Theoretically, it enriches the theoretical framework by elucidating the mediating role of emotional experience and the moderating role of symbolic perception in the influence of digital technology use on sports consumption. Practically, the findings offer substantial guidance for enhancing college students’ engagement in sports consumption activities. It is suggested that policymakers utilize digital technology to foster sports consumption popularity and participation, that universities intensify the convergence of sports with digital technology to boost student involvement, and that families and society at large advocate for healthy sports consumption concepts. Recognizing the symbolic value of sports products and encouraging rational and sustainable sports consumption behaviors are also imperative.

## 5 Conclusion

The research concludes with the following findings: (1) Digital technology use exerts a positive influence on college students’ sports consumption behavior, indicating that engagement with digital platforms and tools can stimulate interest and participation in sports-related activities. (2) The relationship between digital technology use and sports consumption behavior is partially mediated by emotional experience. This suggests that the positive emotions evoked by digital technology contribute to the enhancement of sports consumption behaviors among college students. (3) Symbolic perception moderates the mediating effect of emotional experience on the relationship between digital technology use and sports consumption behavior. This indicates that individual differences in the perception of symbolic value can influence the extent to which emotional experiences translate into sports consumption actions.

## Data Availability

The original contributions presented in this study are included in this article/Supplementary material, further inquiries can be directed to the corresponding author/s.
