# Supplementary material for: The impact of digital technology on sports consumption: evidence from Chinese college students
Source: Front Psychol. 2025 May 20;16:1501327. doi: 10.3389/fpsyg.2025.1501327 (PMC12129995; doi:10.3389/fpsyg.2025.1501327)
Supplement: Supplementary file 1 [file Data_Sheet_1.zip › Raw Data/Common Method Bias Test.AmosOutput]

共同方法偏误.amw


# \\Mac\Home\Desktop\共同方法偏误.amw

## Analysis Summary

## Date and Time

Date: 2024年9月27日

Time: 22:56:08

## Title

共同方法偏误: 2024年9月27日 22:56

## Groups

## Group number 1 (Group number 1)

## Notes for Group (Group number 1)

The model is recursive.

Sample size = 861

## Variable Summary (Group number 1)

## Your model contains the following variables (Group number 1)

Observed, endogenous variables

Usagesituation

Informationquery

Networkconsumption

Emotionalexperience1

Emotionalexperience2

Emotionalexperience3

tangiblesportsconsumptionbehavior1

tangiblesportsconsumptionbehavior2

tangiblesportsconsumptionbehavior3

symbolicperception1

symbolicperception2

symbolicperception3

participatorysportsconsumptionbehavior1

participatorysportsconsumptionbehavior2

participatorysportsconsumptionbehavior3

participatorysportsconsumptionbehavior4

spectatorsportsconsumptionbehavior1

spectatorsportsconsumptionbehavior2

Unobserved, exogenous variables

DTU

e1

e2

e3

EE

e4

e5

e6

TSC

e7

e8

e9

SP

e10

e11

e12

PSC

e13

e14

e15

e16

SSC

e17

e18

CMB

## Variable counts (Group number 1)

|  |  |
| --- | --- |
| Number of variables in your model: | 43 |
| Number of observed variables: | 18 |
| Number of unobserved variables: | 25 |
| Number of exogenous variables: | 25 |
| Number of endogenous variables: | 18 |

## Parameter Summary (Group number 1)

|  | Weights | Covariances | Variances | Means | Intercepts | Total |
| --- | --- | --- | --- | --- | --- | --- |
| Fixed | 24 | 0 | 1 | 0 | 0 | 25 |
| Labeled | 0 | 15 | 0 | 0 | 0 | 15 |
| Unlabeled | 30 | 0 | 24 | 0 | 0 | 54 |
| Total | 54 | 15 | 25 | 0 | 0 | 94 |

## Models

## Default model (Default model)

## Notes for Model (Default model)

## Computation of degrees of freedom (Default model)

|  |  |
| --- | --- |
| Number of distinct sample moments: | 171 |
| Number of distinct parameters to be estimated: | 69 |
| Degrees of freedom (171 - 69): | 102 |

## Result (Default model)

Minimum was achieved

Chi-square = 118.515

Degrees of freedom = 102

Probability level = .126

## Group number 1 (Group number 1 - Default model)

## Estimates (Group number 1 - Default model)

## Scalar Estimates (Group number 1 - Default model)

## Maximum Likelihood Estimates

## Regression Weights: (Group number 1 - Default model)

|  |  |  | Estimate | S.E. | C.R. | P | Label |
| --- | --- | --- | --- | --- | --- | --- | --- |
| Usagesituation | <--- | DTU | 1.000 |  |
| Informationquery | <--- | DTU | 1.019 | .072 | 14.158 | \*\*\* |  |
| Networkconsumption | <--- | DTU | .998 | .073 | 13.720 | \*\*\* |  |
| Emotionalexperience1 | <--- | EE | 1.000 |  |
| Emotionalexperience2 | <--- | EE | 1.123 | .067 | 16.861 | \*\*\* |  |
| Emotionalexperience3 | <--- | EE | .967 | .063 | 15.459 | \*\*\* |  |
| tangiblesportsconsumptionbehavior1 | <--- | TSC | 1.000 |  |
| tangiblesportsconsumptionbehavior2 | <--- | TSC | 1.187 | .146 | 8.147 | \*\*\* |  |
| tangiblesportsconsumptionbehavior3 | <--- | TSC | .947 | .082 | 11.572 | \*\*\* |  |
| symbolicperception1 | <--- | SP | 1.000 |  |
| symbolicperception2 | <--- | SP | 1.150 | .071 | 16.232 | \*\*\* |  |
| symbolicperception3 | <--- | SP | 1.142 | .071 | 16.189 | \*\*\* |  |
| participatorysportsconsumptionbehavior1 | <--- | PSC | 1.000 |  |
| participatorysportsconsumptionbehavior2 | <--- | PSC | .818 | .060 | 13.543 | \*\*\* |  |
| participatorysportsconsumptionbehavior3 | <--- | PSC | .947 | .066 | 14.363 | \*\*\* |  |
| participatorysportsconsumptionbehavior4 | <--- | PSC | .792 | .071 | 11.177 | \*\*\* |  |
| spectatorsportsconsumptionbehavior1 | <--- | SSC | 1.000 |  |
| spectatorsportsconsumptionbehavior2 | <--- | SSC | .902 | .101 | 8.958 | \*\*\* |  |
| Usagesituation | <--- | CMB | .426 | .061 | 6.981 | \*\*\* |  |
| Informationquery | <--- | CMB | .405 | .058 | 6.967 | \*\*\* |  |
| Networkconsumption | <--- | CMB | .435 | .062 | 7.019 | \*\*\* |  |
| Emotionalexperience2 | <--- | CMB | .345 | .071 | 4.846 | \*\*\* |  |
| Emotionalexperience1 | <--- | CMB | .366 | .065 | 5.589 | \*\*\* |  |
| Emotionalexperience3 | <--- | CMB | .191 | .065 | 2.921 | .003 |  |
| symbolicperception1 | <--- | CMB | .260 | .059 | 4.378 | \*\*\* |  |
| symbolicperception2 | <--- | CMB | .456 | .063 | 7.214 | \*\*\* |  |
| symbolicperception3 | <--- | CMB | .459 | .063 | 7.276 | \*\*\* |  |
| tangiblesportsconsumptionbehavior1 | <--- | CMB | .557 | .069 | 8.038 | \*\*\* |  |
| tangiblesportsconsumptionbehavior2 | <--- | CMB | .405 | .072 | 5.657 | \*\*\* |  |
| tangiblesportsconsumptionbehavior3 | <--- | CMB | .708 | .071 | 10.026 | \*\*\* |  |
| participatorysportsconsumptionbehavior1 | <--- | CMB | .319 | .072 | 4.457 | \*\*\* |  |
| participatorysportsconsumptionbehavior2 | <--- | CMB | .469 | .062 | 7.611 | \*\*\* |  |
| participatorysportsconsumptionbehavior3 | <--- | CMB | .356 | .068 | 5.195 | \*\*\* |  |
| participatorysportsconsumptionbehavior4 | <--- | CMB | .647 | .063 | 10.206 | \*\*\* |  |
| spectatorsportsconsumptionbehavior1 | <--- | CMB | .316 | .060 | 5.282 | \*\*\* |  |
| spectatorsportsconsumptionbehavior2 | <--- | CMB | .318 | .057 | 5.619 | \*\*\* |  |

## Standardized Regression Weights: (Group number 1 - Default model)

|  |  |  | Estimate |
| --- | --- | --- | --- |
| Usagesituation | <--- | DTU | .635 |
| Informationquery | <--- | DTU | .713 |
| Networkconsumption | <--- | DTU | .617 |
| Emotionalexperience1 | <--- | EE | .686 |
| Emotionalexperience2 | <--- | EE | .746 |
| Emotionalexperience3 | <--- | EE | .710 |
| tangiblesportsconsumptionbehavior1 | <--- | TSC | .577 |
| tangiblesportsconsumptionbehavior2 | <--- | TSC | .707 |
| tangiblesportsconsumptionbehavior3 | <--- | TSC | .505 |
| symbolicperception1 | <--- | SP | .690 |
| symbolicperception2 | <--- | SP | .720 |
| symbolicperception3 | <--- | SP | .710 |
| participatorysportsconsumptionbehavior1 | <--- | PSC | .744 |
| participatorysportsconsumptionbehavior2 | <--- | PSC | .604 |
| participatorysportsconsumptionbehavior3 | <--- | PSC | .706 |
| participatorysportsconsumptionbehavior4 | <--- | PSC | .537 |
| spectatorsportsconsumptionbehavior1 | <--- | SSC | .759 |
| spectatorsportsconsumptionbehavior2 | <--- | SSC | .705 |
| Usagesituation | <--- | CMB | .390 |
| Informationquery | <--- | CMB | .408 |
| Networkconsumption | <--- | CMB | .387 |
| Emotionalexperience2 | <--- | CMB | .299 |
| Emotionalexperience1 | <--- | CMB | .327 |
| Emotionalexperience3 | <--- | CMB | .183 |
| symbolicperception1 | <--- | CMB | .258 |
| symbolicperception2 | <--- | CMB | .411 |
| symbolicperception3 | <--- | CMB | .412 |
| tangiblesportsconsumptionbehavior1 | <--- | CMB | .552 |
| tangiblesportsconsumptionbehavior2 | <--- | CMB | .414 |
| tangiblesportsconsumptionbehavior3 | <--- | CMB | .648 |
| participatorysportsconsumptionbehavior1 | <--- | CMB | .324 |
| participatorysportsconsumptionbehavior2 | <--- | CMB | .474 |
| participatorysportsconsumptionbehavior3 | <--- | CMB | .363 |
| participatorysportsconsumptionbehavior4 | <--- | CMB | .600 |
| spectatorsportsconsumptionbehavior1 | <--- | CMB | .319 |
| spectatorsportsconsumptionbehavior2 | <--- | CMB | .331 |

## Covariances: (Group number 1 - Default model)

|  |  |  | Estimate | S.E. | C.R. | P | Label |
| --- | --- | --- | --- | --- | --- | --- | --- |
| DTU | <--> | EE | .264 | .045 | 5.941 | \*\*\* | C1 |
| DTU | <--> | TSC | .129 | .047 | 2.717 | .007 | C2 |
| DTU | <--> | PSC | .167 | .046 | 3.648 | \*\*\* | C3 |
| DTU | <--> | SP | .151 | .038 | 4.009 | \*\*\* | C4 |
| DTU | <--> | SSC | .197 | .041 | 4.788 | \*\*\* | C5 |
| EE | <--> | TSC | .190 | .050 | 3.823 | \*\*\* | C6 |
| EE | <--> | PSC | .202 | .045 | 4.535 | \*\*\* | C7 |
| EE | <--> | SP | .235 | .039 | 6.019 | \*\*\* | C8 |
| EE | <--> | SSC | .218 | .041 | 5.334 | \*\*\* | C9 |
| TSC | <--> | PSC | .128 | .052 | 2.443 | .015 | C10 |
| TSC | <--> | SP | .111 | .042 | 2.615 | .009 | C11 |
| TSC | <--> | SSC | .146 | .046 | 3.189 | .001 | C12 |
| SP | <--> | PSC | .159 | .041 | 3.867 | \*\*\* | C13 |
| PSC | <--> | SSC | .208 | .044 | 4.735 | \*\*\* | C14 |
| SP | <--> | SSC | .143 | .036 | 3.935 | \*\*\* | C15 |

## Correlations: (Group number 1 - Default model)

|  |  |  | Estimate |
| --- | --- | --- | --- |
| DTU | <--> | EE | .496 |
| DTU | <--> | TSC | .318 |
| DTU | <--> | PSC | .328 |
| DTU | <--> | SP | .312 |
| DTU | <--> | SSC | .378 |
| EE | <--> | TSC | .424 |
| EE | <--> | PSC | .360 |
| EE | <--> | SP | .442 |
| EE | <--> | SSC | .378 |
| TSC | <--> | PSC | .300 |
| TSC | <--> | SP | .274 |
| TSC | <--> | SSC | .334 |
| SP | <--> | PSC | .314 |
| PSC | <--> | SSC | .379 |
| SP | <--> | SSC | .273 |

## Variances: (Group number 1 - Default model)

|  |  |  | Estimate | S.E. | C.R. | P | Label |
| --- | --- | --- | --- | --- | --- | --- | --- |
| CMB |  |  | 1.000 |  |
| DTU |  |  | .483 | .065 | 7.398 | \*\*\* |  |
| EE |  |  | .588 | .067 | 8.721 | \*\*\* |  |
| TSC |  |  | .340 | .079 | 4.278 | \*\*\* |  |
| SP |  |  | .482 | .054 | 8.977 | \*\*\* |  |
| PSC |  |  | .534 | .065 | 8.284 | \*\*\* |  |
| SSC |  |  | .564 | .077 | 7.344 | \*\*\* |  |
| e1 |  |  | .533 | .036 | 14.992 | \*\*\* |  |
| e2 |  |  | .321 | .029 | 10.946 | \*\*\* |  |
| e3 |  |  | .593 | .038 | 15.682 | \*\*\* |  |
| e4 |  |  | .529 | .035 | 14.954 | \*\*\* |  |
| e5 |  |  | .471 | .039 | 12.209 | \*\*\* |  |
| e6 |  |  | .504 | .037 | 13.770 | \*\*\* |  |
| e7 |  |  | .370 | .026 | 14.068 | \*\*\* |  |
| e8 |  |  | .315 | .043 | 7.267 | \*\*\* |  |
| e9 |  |  | .388 | .031 | 12.420 | \*\*\* |  |
| e10 |  |  | .462 | .031 | 14.781 | \*\*\* |  |
| e11 |  |  | .386 | .031 | 12.363 | \*\*\* |  |
| e12 |  |  | .407 | .032 | 12.880 | \*\*\* |  |
| e13 |  |  | .331 | .030 | 11.064 | \*\*\* |  |
| e14 |  |  | .401 | .024 | 16.706 | \*\*\* |  |
| e15 |  |  | .356 | .027 | 13.403 | \*\*\* |  |
| e16 |  |  | .410 | .032 | 12.906 | \*\*\* |  |
| e17 |  |  | .315 | .056 | 5.650 | \*\*\* |  |
| e18 |  |  | .364 | .046 | 7.915 | \*\*\* |  |

## Squared Multiple Correlations: (Group number 1 - Default model)

|  |  |  | Estimate |
| --- | --- | --- | --- |
| spectatorsportsconsumptionbehavior2 |  |  | .606 |
| spectatorsportsconsumptionbehavior1 |  |  | .678 |
| participatorysportsconsumptionbehavior4 |  |  | .648 |
| participatorysportsconsumptionbehavior3 |  |  | .630 |
| participatorysportsconsumptionbehavior2 |  |  | .590 |
| participatorysportsconsumptionbehavior1 |  |  | .658 |
| symbolicperception3 |  |  | .674 |
| symbolicperception2 |  |  | .687 |
| symbolicperception1 |  |  | .543 |
| tangiblesportsconsumptionbehavior3 |  |  | .675 |
| tangiblesportsconsumptionbehavior2 |  |  | .671 |
| tangiblesportsconsumptionbehavior1 |  |  | .637 |
| Emotionalexperience3 |  |  | .538 |
| Emotionalexperience2 |  |  | .646 |
| Emotionalexperience1 |  |  | .577 |
| Networkconsumption |  |  | .531 |
| Informationquery |  |  | .675 |
| Usagesituation |  |  | .555 |

## Matrices (Group number 1 - Default model)

## Implied (for all variables) Covariances (Group number 1 - Default model)

|  | CMB | SSC | PSC | SP | TSC | EE | DTU | spectatorsportsconsumptionbehavior2 | spectatorsportsconsumptionbehavior1 | participatorysportsconsumptionbehavior4 | participatorysportsconsumptionbehavior3 | participatorysportsconsumptionbehavior2 | participatorysportsconsumptionbehavior1 | symbolicperception3 | symbolicperception2 | symbolicperception1 | tangiblesportsconsumptionbehavior3 | tangiblesportsconsumptionbehavior2 | tangiblesportsconsumptionbehavior1 | Emotionalexperience3 | Emotionalexperience2 | Emotionalexperience1 | Networkconsumption | Informationquery | Usagesituation |
| --- | --- | --- | --- | --- | --- | --- | --- | --- | --- | --- | --- | --- | --- | --- | --- | --- | --- | --- | --- | --- | --- | --- | --- | --- | --- |
| CMB | 1.000 |
| SSC | .000 | .564 |
| PSC | .000 | .208 | .534 |
| SP | .000 | .143 | .159 | .482 |
| TSC | .000 | .146 | .128 | .111 | .340 |
| EE | .000 | .218 | .202 | .235 | .190 | .588 |
| DTU | .000 | .197 | .167 | .151 | .129 | .264 | .483 |
| spectatorsportsconsumptionbehavior2 | .318 | .509 | .188 | .129 | .132 | .197 | .178 | .924 |
| spectatorsportsconsumptionbehavior1 | .316 | .564 | .208 | .143 | .146 | .218 | .197 | .609 | .978 |
| participatorysportsconsumptionbehavior4 | .647 | .165 | .424 | .126 | .101 | .160 | .132 | .355 | .369 | 1.165 |
| participatorysportsconsumptionbehavior3 | .356 | .197 | .506 | .151 | .121 | .191 | .158 | .291 | .309 | .631 | .961 |
| participatorysportsconsumptionbehavior2 | .469 | .170 | .437 | .130 | .105 | .165 | .137 | .303 | .318 | .651 | .581 | .980 |
| participatorysportsconsumptionbehavior1 | .319 | .208 | .534 | .159 | .128 | .202 | .167 | .289 | .309 | .630 | .619 | .587 | .967 |
| symbolicperception3 | .459 | .163 | .182 | .550 | .127 | .269 | .172 | .293 | .308 | .442 | .336 | .364 | .328 | 1.246 |
| symbolicperception2 | .456 | .164 | .183 | .554 | .128 | .271 | .173 | .293 | .308 | .440 | .336 | .364 | .328 | .843 | 1.231 |
| symbolicperception1 | .260 | .143 | .159 | .482 | .111 | .235 | .151 | .211 | .224 | .294 | .243 | .252 | .242 | .670 | .673 | 1.011 |
| tangiblesportsconsumptionbehavior3 | .708 | .139 | .121 | .105 | .322 | .180 | .122 | .350 | .362 | .555 | .367 | .432 | .347 | .445 | .444 | .289 | 1.195 |
| tangiblesportsconsumptionbehavior2 | .405 | .174 | .152 | .132 | .404 | .225 | .153 | .286 | .302 | .383 | .288 | .314 | .281 | .336 | .336 | .237 | .669 | .958 |
| tangiblesportsconsumptionbehavior1 | .557 | .146 | .128 | .111 | .340 | .190 | .129 | .309 | .322 | .462 | .320 | .366 | .306 | .383 | .382 | .256 | .717 | .629 | 1.021 |
| Emotionalexperience3 | .191 | .211 | .195 | .228 | .183 | .569 | .256 | .251 | .271 | .278 | .253 | .249 | .256 | .348 | .349 | .277 | .309 | .295 | .290 | 1.091 |
| Emotionalexperience2 | .345 | .245 | .227 | .264 | .213 | .661 | .297 | .331 | .354 | .403 | .338 | .348 | .337 | .460 | .461 | .354 | .446 | .393 | .406 | .705 | 1.333 |
| Emotionalexperience1 | .366 | .218 | .202 | .235 | .190 | .588 | .264 | .313 | .333 | .397 | .321 | .337 | .319 | .437 | .437 | .330 | .439 | .373 | .393 | .639 | .787 | 1.250 |
| Networkconsumption | .435 | .197 | .166 | .150 | .129 | .264 | .482 | .316 | .334 | .414 | .312 | .340 | .305 | .372 | .371 | .263 | .430 | .329 | .371 | .338 | .447 | .423 | 1.263 |
| Informationquery | .405 | .201 | .170 | .154 | .131 | .270 | .492 | .310 | .329 | .397 | .305 | .329 | .299 | .361 | .361 | .259 | .411 | .320 | .357 | .338 | .443 | .418 | .667 | .986 |
| Usagesituation | .426 | .197 | .167 | .151 | .129 | .264 | .483 | .313 | .332 | .408 | .310 | .337 | .303 | .368 | .368 | .261 | .424 | .326 | .366 | .337 | .444 | .420 | .667 | .665 | 1.197 |

## Implied (for all variables) Correlations (Group number 1 - Default model)

|  | CMB | SSC | PSC | SP | TSC | EE | DTU | spectatorsportsconsumptionbehavior2 | spectatorsportsconsumptionbehavior1 | participatorysportsconsumptionbehavior4 | participatorysportsconsumptionbehavior3 | participatorysportsconsumptionbehavior2 | participatorysportsconsumptionbehavior1 | symbolicperception3 | symbolicperception2 | symbolicperception1 | tangiblesportsconsumptionbehavior3 | tangiblesportsconsumptionbehavior2 | tangiblesportsconsumptionbehavior1 | Emotionalexperience3 | Emotionalexperience2 | Emotionalexperience1 | Networkconsumption | Informationquery | Usagesituation |
| --- | --- | --- | --- | --- | --- | --- | --- | --- | --- | --- | --- | --- | --- | --- | --- | --- | --- | --- | --- | --- | --- | --- | --- | --- | --- |
| CMB | 1.000 |
| SSC | .000 | 1.000 |
| PSC | .000 | .379 | 1.000 |
| SP | .000 | .273 | .314 | 1.000 |
| TSC | .000 | .334 | .300 | .274 | 1.000 |
| EE | .000 | .378 | .360 | .442 | .424 | 1.000 |
| DTU | .000 | .378 | .328 | .312 | .318 | .496 | 1.000 |
| spectatorsportsconsumptionbehavior2 | .331 | .705 | .267 | .193 | .236 | .267 | .266 | 1.000 |
| spectatorsportsconsumptionbehavior1 | .319 | .759 | .288 | .208 | .254 | .287 | .287 | .641 | 1.000 |
| participatorysportsconsumptionbehavior4 | .600 | .203 | .537 | .168 | .161 | .193 | .176 | .342 | .346 | 1.000 |
| participatorysportsconsumptionbehavior3 | .363 | .267 | .706 | .221 | .212 | .254 | .232 | .309 | .319 | .597 | 1.000 |
| participatorysportsconsumptionbehavior2 | .474 | .229 | .604 | .190 | .182 | .218 | .199 | .318 | .325 | .609 | .599 | 1.000 |
| participatorysportsconsumptionbehavior1 | .324 | .282 | .744 | .233 | .223 | .268 | .244 | .306 | .317 | .594 | .642 | .603 | 1.000 |
| symbolicperception3 | .412 | .194 | .223 | .710 | .195 | .314 | .222 | .273 | .279 | .366 | .307 | .330 | .299 | 1.000 |
| symbolicperception2 | .411 | .197 | .226 | .720 | .197 | .318 | .225 | .275 | .280 | .368 | .308 | .331 | .301 | .680 | 1.000 |
| symbolicperception1 | .258 | .189 | .217 | .690 | .189 | .305 | .216 | .219 | .226 | .271 | .247 | .253 | .245 | .597 | .603 | 1.000 |
| tangiblesportsconsumptionbehavior3 | .648 | .169 | .152 | .139 | .505 | .214 | .161 | .333 | .335 | .470 | .342 | .399 | .323 | .365 | .366 | .263 | 1.000 |
| tangiblesportsconsumptionbehavior2 | .414 | .236 | .212 | .194 | .707 | .300 | .225 | .303 | .311 | .362 | .300 | .325 | .292 | .308 | .309 | .241 | .625 | 1.000 |
| tangiblesportsconsumptionbehavior1 | .552 | .193 | .173 | .158 | .577 | .245 | .184 | .318 | .322 | .424 | .323 | .366 | .308 | .339 | .340 | .252 | .649 | .636 | 1.000 |
| Emotionalexperience3 | .183 | .269 | .256 | .314 | .301 | .710 | .352 | .250 | .262 | .247 | .247 | .241 | .250 | .298 | .301 | .264 | .271 | .289 | .275 | 1.000 |
| Emotionalexperience2 | .299 | .282 | .269 | .330 | .317 | .746 | .370 | .298 | .310 | .324 | .298 | .304 | .297 | .357 | .360 | .305 | .354 | .348 | .348 | .585 | 1.000 |
| Emotionalexperience1 | .327 | .259 | .247 | .303 | .291 | .686 | .340 | .291 | .301 | .329 | .293 | .304 | .290 | .350 | .352 | .294 | .359 | .341 | .348 | .547 | .610 | 1.000 |
| Networkconsumption | .387 | .233 | .203 | .193 | .196 | .306 | .617 | .292 | .300 | .341 | .284 | .306 | .276 | .296 | .298 | .233 | .350 | .299 | .327 | .288 | .344 | .337 | 1.000 |
| Informationquery | .408 | .269 | .234 | .223 | .227 | .354 | .713 | .325 | .335 | .370 | .313 | .335 | .306 | .326 | .328 | .259 | .379 | .329 | .356 | .326 | .386 | .376 | .598 | 1.000 |
| Usagesituation | .390 | .240 | .209 | .198 | .202 | .315 | .635 | .298 | .306 | .346 | .289 | .311 | .281 | .301 | .303 | .238 | .355 | .304 | .332 | .295 | .352 | .343 | .543 | .612 | 1.000 |

## Minimization History (Default model)

| Iteration |  | Negative eigenvalues | Condition # | Smallest eigenvalue | Diameter | F | NTries | Ratio |
| --- | --- | --- | --- | --- | --- | --- | --- | --- |
| 0 | e | 26 |  | -1.683 | 9999.000 | 7250.564 | 0 | 9999.000 |
| 1 | e | 26 |  | -.157 | 1.761 | 4674.238 | 19 | .342 |
| 2 | e\* | 3 |  | -1.568 | 3.024 | 1213.055 | 5 | .632 |
| 3 | e | 3 |  | -.120 | .728 | 569.038 | 8 | .565 |
| 4 | e | 2 |  | -.006 | 1.248 | 243.456 | 6 | .594 |
| 5 | e | 1 |  | -.005 | .500 | 152.210 | 5 | .943 |
| 6 | e | 0 | 778.442 |  | .384 | 141.762 | 6 | .877 |
| 7 | e | 1 |  | -.008 | .195 | 139.016 | 1 | 1.182 |
| 8 | e | 0 | 637.549 |  | .300 | 137.437 | 5 | .589 |
| 9 | e | 0 | 409.910 |  | .512 | 132.301 | 1 | 1.005 |
| 10 | e | 0 | 390.445 |  | .340 | 120.111 | 1 | 1.022 |
| 11 | e | 0 | 531.583 |  | .076 | 118.538 | 1 | .975 |
| 12 | e | 0 | 521.519 |  | .023 | 118.515 | 1 | 1.008 |
| 13 | e | 0 | 506.506 |  | .001 | 118.515 | 1 | 1.001 |

## Model44 (Model44)

## Notes for Model (Model44)

## Computation of degrees of freedom (Model44)

|  |  |
| --- | --- |
| Number of distinct sample moments: | 171 |
| Number of distinct parameters to be estimated: | 54 |
| Degrees of freedom (171 - 54): | 117 |

## Result (Model44)

Minimum was achieved

Chi-square = 135.973

Degrees of freedom = 117

Probability level = .111

## Group number 1 (Group number 1 - Model44)

## Estimates (Group number 1 - Model44)

## Scalar Estimates (Group number 1 - Model44)

## Maximum Likelihood Estimates

## Regression Weights: (Group number 1 - Model44)

|  |  |  | Estimate | S.E. | C.R. | P | Label |
| --- | --- | --- | --- | --- | --- | --- | --- |
| Usagesituation | <--- | DTU | 1.000 |  |
| Informationquery | <--- | DTU | 1.001 | .042 | 23.887 | \*\*\* |  |
| Networkconsumption | <--- | DTU | .988 | .048 | 20.720 | \*\*\* |  |
| Emotionalexperience1 | <--- | EE | 1.000 |  |
| Emotionalexperience2 | <--- | EE | 1.094 | .046 | 23.814 | \*\*\* |  |
| Emotionalexperience3 | <--- | EE | .888 | .043 | 20.620 | \*\*\* |  |
| tangiblesportsconsumptionbehavior1 | <--- | TSC | 1.000 |  |
| tangiblesportsconsumptionbehavior2 | <--- | TSC | .932 | .040 | 23.572 | \*\*\* |  |
| tangiblesportsconsumptionbehavior3 | <--- | TSC | 1.047 | .046 | 22.846 | \*\*\* |  |
| symbolicperception1 | <--- | SP | 1.000 |  |
| symbolicperception2 | <--- | SP | 1.266 | .053 | 23.692 | \*\*\* |  |
| symbolicperception3 | <--- | SP | 1.250 | .054 | 23.036 | \*\*\* |  |
| participatorysportsconsumptionbehavior1 | <--- | PSC | 1.000 |  |
| participatorysportsconsumptionbehavior2 | <--- | PSC | .962 | .046 | 21.123 | \*\*\* |  |
| participatorysportsconsumptionbehavior3 | <--- | PSC | 1.054 | .049 | 21.372 | \*\*\* |  |
| participatorysportsconsumptionbehavior4 | <--- | PSC | 1.034 | .090 | 11.463 | \*\*\* |  |
| spectatorsportsconsumptionbehavior1 | <--- | SSC | 1.000 |  |
| spectatorsportsconsumptionbehavior2 | <--- | SSC | .949 | .053 | 17.842 | \*\*\* |  |
| Usagesituation | <--- | CMB | .192 | .072 | 2.686 | .007 |  |
| Informationquery | <--- | CMB | .140 | .068 | 2.063 | .039 |  |
| Networkconsumption | <--- | CMB | .205 | .073 | 2.806 | .005 |  |
| Emotionalexperience2 | <--- | CMB | .131 | .076 | 1.731 | .083 |  |
| Emotionalexperience1 | <--- | CMB | .157 | .071 | 2.210 | .027 |  |
| Emotionalexperience3 | <--- | CMB | .035 | .065 | .543 | .587 |  |
| symbolicperception1 | <--- | CMB | .090 | .063 | 1.440 | .150 |  |
| symbolicperception2 | <--- | CMB | .145 | .074 | 1.956 | .051 |  |
| symbolicperception3 | <--- | CMB | .194 | .075 | 2.600 | .009 |  |
| tangiblesportsconsumptionbehavior1 | <--- | CMB | .231 | .072 | 3.182 | .001 |  |
| tangiblesportsconsumptionbehavior2 | <--- | CMB | .161 | .069 | 2.348 | .019 |  |
| tangiblesportsconsumptionbehavior3 | <--- | CMB | .315 | .081 | 3.903 | \*\*\* |  |
| participatorysportsconsumptionbehavior1 | <--- | CMB | .038 | .099 | .381 | .703 |  |
| participatorysportsconsumptionbehavior2 | <--- | CMB | .135 | .098 | 1.386 | .166 |  |
| participatorysportsconsumptionbehavior3 | <--- | CMB | -.072 | .109 | -.658 | .510 |  |
| participatorysportsconsumptionbehavior4 | <--- | CMB | .549 | .127 | 4.311 | \*\*\* |  |
| spectatorsportsconsumptionbehavior1 | <--- | CMB | .144 | .066 | 2.175 | .030 |  |
| spectatorsportsconsumptionbehavior2 | <--- | CMB | .120 | .064 | 1.879 | .060 |  |

## Standardized Regression Weights: (Group number 1 - Model44)

|  |  |  | Estimate |
| --- | --- | --- | --- |
| Usagesituation | <--- | DTU | .736 |
| Informationquery | <--- | DTU | .814 |
| Networkconsumption | <--- | DTU | .712 |
| Emotionalexperience1 | <--- | EE | .757 |
| Emotionalexperience2 | <--- | EE | .802 |
| Emotionalexperience3 | <--- | EE | .721 |
| tangiblesportsconsumptionbehavior1 | <--- | TSC | .788 |
| tangiblesportsconsumptionbehavior2 | <--- | TSC | .765 |
| tangiblesportsconsumptionbehavior3 | <--- | TSC | .769 |
| symbolicperception1 | <--- | SP | .719 |
| symbolicperception2 | <--- | SP | .825 |
| symbolicperception3 | <--- | SP | .809 |
| participatorysportsconsumptionbehavior1 | <--- | PSC | .787 |
| participatorysportsconsumptionbehavior2 | <--- | PSC | .753 |
| participatorysportsconsumptionbehavior3 | <--- | PSC | .832 |
| participatorysportsconsumptionbehavior4 | <--- | PSC | .743 |
| spectatorsportsconsumptionbehavior1 | <--- | SSC | .802 |
| spectatorsportsconsumptionbehavior2 | <--- | SSC | .785 |
| Usagesituation | <--- | CMB | .173 |
| Informationquery | <--- | CMB | .139 |
| Networkconsumption | <--- | CMB | .181 |
| Emotionalexperience2 | <--- | CMB | .112 |
| Emotionalexperience1 | <--- | CMB | .140 |
| Emotionalexperience3 | <--- | CMB | .034 |
| symbolicperception1 | <--- | CMB | .089 |
| symbolicperception2 | <--- | CMB | .130 |
| symbolicperception3 | <--- | CMB | .172 |
| tangiblesportsconsumptionbehavior1 | <--- | CMB | .223 |
| tangiblesportsconsumptionbehavior2 | <--- | CMB | .163 |
| tangiblesportsconsumptionbehavior3 | <--- | CMB | .284 |
| participatorysportsconsumptionbehavior1 | <--- | CMB | .038 |
| participatorysportsconsumptionbehavior2 | <--- | CMB | .136 |
| participatorysportsconsumptionbehavior3 | <--- | CMB | -.073 |
| participatorysportsconsumptionbehavior4 | <--- | CMB | .505 |
| spectatorsportsconsumptionbehavior1 | <--- | CMB | .144 |
| spectatorsportsconsumptionbehavior2 | <--- | CMB | .124 |

## Covariances: (Group number 1 - Model44)

|  |  |  | Estimate | S.E. | C.R. | P | Label |
| --- | --- | --- | --- | --- | --- | --- | --- |
| DTU | <--> | EE | .410 |  |
| DTU | <--> | TSC | .367 |  |
| DTU | <--> | PSC | .327 |  |
| DTU | <--> | SP | .281 |  |
| DTU | <--> | SSC | .330 |  |
| EE | <--> | TSC | .377 |  |
| EE | <--> | PSC | .317 |  |
| EE | <--> | SP | .332 |  |
| EE | <--> | SSC | .323 |  |
| TSC | <--> | PSC | .356 |  |
| TSC | <--> | SP | .296 |  |
| TSC | <--> | SSC | .326 |  |
| SP | <--> | PSC | .272 |  |
| PSC | <--> | SSC | .317 |  |
| SP | <--> | SSC | .238 |  |

## Correlations: (Group number 1 - Model44)

|  |  |  | Estimate |
| --- | --- | --- | --- |
| DTU | <--> | EE | .588 |
| DTU | <--> | TSC | .552 |
| DTU | <--> | PSC | .513 |
| DTU | <--> | SP | .473 |
| DTU | <--> | SSC | .504 |
| EE | <--> | TSC | .543 |
| EE | <--> | PSC | .476 |
| EE | <--> | SP | .535 |
| EE | <--> | SSC | .473 |
| TSC | <--> | PSC | .560 |
| TSC | <--> | SP | .500 |
| TSC | <--> | SSC | .500 |
| SP | <--> | PSC | .479 |
| PSC | <--> | SSC | .507 |
| SP | <--> | SSC | .408 |

## Variances: (Group number 1 - Model44)

|  |  |  | Estimate | S.E. | C.R. | P | Label |
| --- | --- | --- | --- | --- | --- | --- | --- |
| CMB |  |  | 1.000 |  |
| DTU |  |  | .668 | .032 | 20.747 | \*\*\* |  |
| EE |  |  | .728 | .035 | 20.714 | \*\*\* |  |
| TSC |  |  | .662 | .030 | 22.316 | \*\*\* |  |
| SP |  |  | .530 | .029 | 18.236 | \*\*\* |  |
| PSC |  |  | .609 | .032 | 19.040 | \*\*\* |  |
| SSC |  |  | .642 | .038 | 17.005 | \*\*\* |  |
| e1 |  |  | .529 | .034 | 15.597 | \*\*\* |  |
| e2 |  |  | .322 | .027 | 11.964 | \*\*\* |  |
| e3 |  |  | .592 | .037 | 16.075 | \*\*\* |  |
| e4 |  |  | .516 | .035 | 14.877 | \*\*\* |  |
| e5 |  |  | .465 | .036 | 12.793 | \*\*\* |  |
| e6 |  |  | .530 | .034 | 15.714 | \*\*\* |  |
| e7 |  |  | .352 | .025 | 13.934 | \*\*\* |  |
| e8 |  |  | .382 | .025 | 15.009 | \*\*\* |  |
| e9 |  |  | .402 | .029 | 13.815 | \*\*\* |  |
| e10 |  |  | .486 | .029 | 16.796 | \*\*\* |  |
| e11 |  |  | .379 | .031 | 12.146 | \*\*\* |  |
| e12 |  |  | .399 | .031 | 12.749 | \*\*\* |  |
| e13 |  |  | .372 | .027 | 13.838 | \*\*\* |  |
| e14 |  |  | .411 | .025 | 16.777 | \*\*\* |  |
| e15 |  |  | .296 | .037 | 7.960 | \*\*\* |  |
| e16 |  |  | .228 | .084 | 2.731 | .006 |  |
| e17 |  |  | .335 | .036 | 9.351 | \*\*\* |  |
| e18 |  |  | .346 | .033 | 10.350 | \*\*\* |  |

## Squared Multiple Correlations: (Group number 1 - Model44)

|  |  |  | Estimate |
| --- | --- | --- | --- |
| spectatorsportsconsumptionbehavior2 |  |  | .631 |
| spectatorsportsconsumptionbehavior1 |  |  | .664 |
| participatorysportsconsumptionbehavior4 |  |  | .807 |
| participatorysportsconsumptionbehavior3 |  |  | .697 |
| participatorysportsconsumptionbehavior2 |  |  | .586 |
| participatorysportsconsumptionbehavior1 |  |  | .621 |
| symbolicperception3 |  |  | .684 |
| symbolicperception2 |  |  | .697 |
| symbolicperception1 |  |  | .525 |
| tangiblesportsconsumptionbehavior3 |  |  | .673 |
| tangiblesportsconsumptionbehavior2 |  |  | .612 |
| tangiblesportsconsumptionbehavior1 |  |  | .671 |
| Emotionalexperience3 |  |  | .521 |
| Emotionalexperience2 |  |  | .656 |
| Emotionalexperience1 |  |  | .593 |
| Networkconsumption |  |  | .539 |
| Informationquery |  |  | .681 |
| Usagesituation |  |  | .571 |

## Matrices (Group number 1 - Model44)

## Implied (for all variables) Covariances (Group number 1 - Model44)

|  | CMB | SSC | PSC | SP | TSC | EE | DTU | spectatorsportsconsumptionbehavior2 | spectatorsportsconsumptionbehavior1 | participatorysportsconsumptionbehavior4 | participatorysportsconsumptionbehavior3 | participatorysportsconsumptionbehavior2 | participatorysportsconsumptionbehavior1 | symbolicperception3 | symbolicperception2 | symbolicperception1 | tangiblesportsconsumptionbehavior3 | tangiblesportsconsumptionbehavior2 | tangiblesportsconsumptionbehavior1 | Emotionalexperience3 | Emotionalexperience2 | Emotionalexperience1 | Networkconsumption | Informationquery | Usagesituation |
| --- | --- | --- | --- | --- | --- | --- | --- | --- | --- | --- | --- | --- | --- | --- | --- | --- | --- | --- | --- | --- | --- | --- | --- | --- | --- |
| CMB | 1.000 |
| SSC | .000 | .642 |
| PSC | .000 | .317 | .609 |
| SP | .000 | .238 | .272 | .530 |
| TSC | .000 | .326 | .356 | .296 | .662 |
| EE | .000 | .323 | .317 | .332 | .377 | .728 |
| DTU | .000 | .330 | .327 | .281 | .367 | .410 | .668 |
| spectatorsportsconsumptionbehavior2 | .120 | .609 | .301 | .226 | .309 | .307 | .313 | .939 |
| spectatorsportsconsumptionbehavior1 | .144 | .642 | .317 | .238 | .326 | .323 | .330 | .626 | .998 |
| participatorysportsconsumptionbehavior4 | .549 | .328 | .630 | .281 | .368 | .328 | .338 | .377 | .407 | 1.181 |
| participatorysportsconsumptionbehavior3 | -.072 | .334 | .642 | .287 | .375 | .334 | .345 | .309 | .324 | .625 | .978 |
| participatorysportsconsumptionbehavior2 | .135 | .305 | .586 | .262 | .343 | .305 | .315 | .306 | .325 | .681 | .608 | .994 |
| participatorysportsconsumptionbehavior1 | .038 | .317 | .609 | .272 | .356 | .317 | .327 | .305 | .322 | .651 | .639 | .591 | .983 |
| symbolicperception3 | .194 | .297 | .340 | .662 | .370 | .415 | .351 | .306 | .325 | .458 | .344 | .353 | .347 | 1.264 |
| symbolicperception2 | .145 | .301 | .344 | .671 | .375 | .420 | .356 | .303 | .322 | .436 | .353 | .351 | .350 | .866 | 1.249 |
| symbolicperception1 | .090 | .238 | .272 | .530 | .296 | .332 | .281 | .237 | .251 | .331 | .280 | .274 | .275 | .679 | .684 | 1.024 |
| tangiblesportsconsumptionbehavior3 | .315 | .341 | .373 | .310 | .694 | .395 | .384 | .362 | .387 | .558 | .370 | .401 | .385 | .448 | .438 | .338 | 1.227 |
| tangiblesportsconsumptionbehavior2 | .161 | .304 | .332 | .276 | .618 | .352 | .342 | .308 | .327 | .432 | .338 | .341 | .338 | .376 | .373 | .291 | .698 | .984 |
| tangiblesportsconsumptionbehavior1 | .231 | .326 | .356 | .296 | .662 | .377 | .367 | .337 | .359 | .495 | .359 | .374 | .365 | .415 | .408 | .317 | .766 | .655 | 1.067 |
| Emotionalexperience3 | .035 | .287 | .281 | .295 | .335 | .646 | .364 | .276 | .292 | .311 | .294 | .276 | .283 | .375 | .378 | .298 | .362 | .318 | .343 | 1.105 |
| Emotionalexperience2 | .131 | .353 | .347 | .363 | .413 | .796 | .449 | .351 | .372 | .431 | .356 | .351 | .352 | .479 | .479 | .375 | .473 | .406 | .443 | .712 | 1.354 |
| Emotionalexperience1 | .157 | .323 | .317 | .332 | .377 | .728 | .410 | .325 | .346 | .414 | .323 | .326 | .323 | .445 | .443 | .346 | .444 | .377 | .413 | .652 | .817 | 1.268 |
| Networkconsumption | .205 | .326 | .323 | .278 | .362 | .405 | .659 | .334 | .355 | .447 | .326 | .339 | .331 | .387 | .381 | .296 | .444 | .371 | .410 | .367 | .470 | .437 | 1.285 |
| Informationquery | .140 | .330 | .327 | .281 | .367 | .410 | .668 | .330 | .350 | .415 | .335 | .334 | .332 | .378 | .376 | .294 | .429 | .365 | .400 | .369 | .467 | .432 | .688 | 1.010 |
| Usagesituation | .192 | .330 | .327 | .281 | .367 | .410 | .668 | .336 | .358 | .444 | .331 | .341 | .334 | .388 | .384 | .298 | .445 | .373 | .411 | .371 | .474 | .440 | .699 | .695 | 1.233 |

## Implied (for all variables) Correlations (Group number 1 - Model44)

|  | CMB | SSC | PSC | SP | TSC | EE | DTU | spectatorsportsconsumptionbehavior2 | spectatorsportsconsumptionbehavior1 | participatorysportsconsumptionbehavior4 | participatorysportsconsumptionbehavior3 | participatorysportsconsumptionbehavior2 | participatorysportsconsumptionbehavior1 | symbolicperception3 | symbolicperception2 | symbolicperception1 | tangiblesportsconsumptionbehavior3 | tangiblesportsconsumptionbehavior2 | tangiblesportsconsumptionbehavior1 | Emotionalexperience3 | Emotionalexperience2 | Emotionalexperience1 | Networkconsumption | Informationquery | Usagesituation |
| --- | --- | --- | --- | --- | --- | --- | --- | --- | --- | --- | --- | --- | --- | --- | --- | --- | --- | --- | --- | --- | --- | --- | --- | --- | --- |
| CMB | 1.000 |
| SSC | .000 | 1.000 |
| PSC | .000 | .507 | 1.000 |
| SP | .000 | .408 | .479 | 1.000 |
| TSC | .000 | .500 | .560 | .500 | 1.000 |
| EE | .000 | .473 | .476 | .535 | .543 | 1.000 |
| DTU | .000 | .504 | .513 | .473 | .552 | .588 | 1.000 |
| spectatorsportsconsumptionbehavior2 | .124 | .785 | .398 | .320 | .392 | .371 | .396 | 1.000 |
| spectatorsportsconsumptionbehavior1 | .144 | .802 | .407 | .327 | .401 | .379 | .404 | .647 | 1.000 |
| participatorysportsconsumptionbehavior4 | .505 | .377 | .743 | .356 | .416 | .354 | .381 | .358 | .375 | 1.000 |
| participatorysportsconsumptionbehavior3 | -.073 | .422 | .832 | .398 | .466 | .396 | .427 | .322 | .328 | .581 | 1.000 |
| participatorysportsconsumptionbehavior2 | .136 | .382 | .753 | .361 | .422 | .359 | .386 | .317 | .326 | .628 | .617 | 1.000 |
| participatorysportsconsumptionbehavior1 | .038 | .399 | .787 | .377 | .441 | .375 | .404 | .318 | .326 | .604 | .652 | .598 | 1.000 |
| symbolicperception3 | .172 | .330 | .387 | .809 | .404 | .433 | .382 | .281 | .290 | .375 | .310 | .315 | .312 | 1.000 |
| symbolicperception2 | .130 | .337 | .395 | .825 | .412 | .441 | .390 | .280 | .289 | .359 | .319 | .315 | .316 | .690 | 1.000 |
| symbolicperception1 | .089 | .294 | .344 | .719 | .359 | .385 | .340 | .241 | .248 | .301 | .280 | .272 | .274 | .597 | .605 | 1.000 |
| tangiblesportsconsumptionbehavior3 | .284 | .385 | .431 | .384 | .769 | .418 | .425 | .337 | .349 | .464 | .338 | .363 | .350 | .360 | .354 | .302 | 1.000 |
| tangiblesportsconsumptionbehavior2 | .163 | .383 | .429 | .382 | .765 | .415 | .422 | .320 | .330 | .401 | .345 | .345 | .344 | .337 | .336 | .289 | .635 | 1.000 |
| tangiblesportsconsumptionbehavior1 | .223 | .394 | .442 | .394 | .788 | .428 | .435 | .337 | .348 | .441 | .351 | .363 | .356 | .357 | .354 | .303 | .670 | .639 | 1.000 |
| Emotionalexperience3 | .034 | .341 | .343 | .385 | .391 | .721 | .424 | .271 | .278 | .272 | .283 | .263 | .271 | .318 | .322 | .280 | .311 | .305 | .316 | 1.000 |
| Emotionalexperience2 | .112 | .379 | .382 | .429 | .436 | .802 | .472 | .311 | .320 | .340 | .310 | .303 | .305 | .366 | .368 | .319 | .367 | .351 | .368 | .582 | 1.000 |
| Emotionalexperience1 | .140 | .358 | .361 | .405 | .411 | .757 | .446 | .298 | .307 | .338 | .290 | .291 | .289 | .352 | .352 | .304 | .356 | .337 | .355 | .551 | .623 | 1.000 |
| Networkconsumption | .181 | .359 | .365 | .336 | .393 | .419 | .712 | .304 | .314 | .363 | .291 | .300 | .294 | .303 | .301 | .258 | .354 | .330 | .350 | .308 | .356 | .342 | 1.000 |
| Informationquery | .139 | .410 | .417 | .384 | .449 | .479 | .814 | .339 | .349 | .380 | .337 | .333 | .334 | .335 | .335 | .289 | .385 | .366 | .385 | .350 | .400 | .382 | .604 | 1.000 |
| Usagesituation | .173 | .371 | .377 | .348 | .406 | .433 | .736 | .313 | .322 | .368 | .301 | .308 | .304 | .311 | .309 | .265 | .362 | .339 | .358 | .318 | .367 | .352 | .555 | .623 | 1.000 |

## Minimization History (Model44)

| Iteration |  | Negative eigenvalues | Condition # | Smallest eigenvalue | Diameter | F | NTries | Ratio |
| --- | --- | --- | --- | --- | --- | --- | --- | --- |
| 0 | e | 14 |  | -1.657 | 9999.000 | 6829.010 | 0 | 9999.000 |
| 1 | e | 12 |  | -.215 | 1.960 | 4649.654 | 20 | .269 |
| 2 | e | 1 |  | -.075 | 2.646 | 773.569 | 5 | .852 |
| 3 | e | 3 |  | -.130 | .720 | 405.075 | 4 | .780 |
| 4 | e | 1 |  | -.035 | .893 | 208.458 | 9 | .811 |
| 5 | e | 0 | 73.946 |  | .565 | 156.106 | 5 | .959 |
| 6 | e | 0 | 97.566 |  | .504 | 139.983 | 1 | 1.034 |
| 7 | e | 0 | 1558.181 |  | .199 | 137.618 | 1 | 1.110 |
| 8 | e | 0 | 206.829 |  | .115 | 136.898 | 5 | .000 |
| 9 | e | 0 | 266.382 |  | .215 | 136.845 | 1 | .056 |
| 10 | e | 0 | 375.885 |  | .069 | 136.062 | 1 | .880 |
| 11 | e | 0 | 281.840 |  | .078 | 136.012 | 1 | .464 |
| 12 | e | 0 | 306.761 |  | .022 | 135.974 | 1 | 1.064 |
| 13 | e | 0 | 313.046 |  | .005 | 135.973 | 1 | 1.020 |
| 14 | e | 0 | 312.397 |  | .000 | 135.973 | 1 | 1.001 |

## Model Fit Summary

## CMIN

| Model | NPAR | CMIN | DF | P | CMIN/DF |
| --- | --- | --- | --- | --- | --- |
| Default model | 69 | 118.515 | 102 | .126 | 1.162 |
| Model44 | 54 | 135.973 | 117 | .111 | 1.162 |
| Saturated model | 171 | .000 | 0 |
| Independence model | 18 | 7330.667 | 153 | .000 | 47.913 |

## RMR, GFI

| Model | RMR | GFI | AGFI | PGFI |
| --- | --- | --- | --- | --- |
| Default model | .017 | .985 | .975 | .588 |
| Model44 | .031 | .983 | .975 | .672 |
| Saturated model | .000 | 1.000 |  |  |
| Independence model | .381 | .306 | .224 | .274 |

## Baseline Comparisons

| Model | NFI Delta1 | RFI rho1 | IFI Delta2 | TLI rho2 | CFI |
| --- | --- | --- | --- | --- | --- |
| Default model | .984 | .976 | .998 | .997 | .998 |
| Model44 | .981 | .976 | .997 | .997 | .997 |
| Saturated model | 1.000 |  | 1.000 |  | 1.000 |
| Independence model | .000 | .000 | .000 | .000 | .000 |

## Parsimony-Adjusted Measures

| Model | PRATIO | PNFI | PCFI |
| --- | --- | --- | --- |
| Default model | .667 | .656 | .665 |
| Model44 | .765 | .751 | .763 |
| Saturated model | .000 | .000 | .000 |
| Independence model | 1.000 | .000 | .000 |

## NCP

| Model | NCP | LO 90 | HI 90 |
| --- | --- | --- | --- |
| Default model | 16.515 | .000 | 47.731 |
| Model44 | 18.973 | .000 | 52.110 |
| Saturated model | .000 | .000 | .000 |
| Independence model | 7177.667 | 6900.649 | 7461.007 |

## FMIN

| Model | FMIN | F0 | LO 90 | HI 90 |
| --- | --- | --- | --- | --- |
| Default model | .138 | .019 | .000 | .056 |
| Model44 | .158 | .022 | .000 | .061 |
| Saturated model | .000 | .000 | .000 | .000 |
| Independence model | 8.524 | 8.346 | 8.024 | 8.676 |

## RMSEA

| Model | RMSEA | LO 90 | HI 90 | PCLOSE |
| --- | --- | --- | --- | --- |
| Default model | .014 | .000 | .023 | 1.000 |
| Model44 | .014 | .000 | .023 | 1.000 |
| Independence model | .234 | .229 | .238 | .000 |

## AIC

| Model | AIC | BCC | BIC | CAIC |
| --- | --- | --- | --- | --- |
| Default model | 256.515 | 259.633 | 584.824 | 653.824 |
| Model44 | 243.973 | 246.413 | 500.910 | 554.910 |
| Saturated model | 342.000 | 349.727 | 1155.634 | 1326.634 |
| Independence model | 7366.667 | 7367.480 | 7452.313 | 7470.313 |

## ECVI

| Model | ECVI | LO 90 | HI 90 | MECVI |
| --- | --- | --- | --- | --- |
| Default model | .298 | .279 | .335 | .302 |
| Model44 | .284 | .262 | .322 | .287 |
| Saturated model | .398 | .398 | .398 | .407 |
| Independence model | 8.566 | 8.244 | 8.895 | 8.567 |

## HOELTER

| Model | HOELTER .05 | HOELTER .01 |
| --- | --- | --- |
| Default model | 919 | 1003 |
| Model44 | 907 | 984 |
| Independence model | 22 | 24 |

## Nested Model Comparisons

## Assuming model Default model to be correct:

|  |  |  |  |  |  |  |  |
| --- | --- | --- | --- | --- | --- | --- | --- |
| Model | DF | CMIN | P | NFI Delta-1 | IFI Delta-2 | RFI rho-1 | TLI rho2 |
| Model44 | 15 | 17.458 | .292 | .002 | .002 | .000 | .000 |

## Execution time summary

|  |  |
| --- | --- |
| Minimization: | .106 |
| Miscellaneous: | .972 |
| Bootstrap: | .000 |
| Total: | 1.078 |
