# Supplementary material for: The impact of digital technology on sports consumption: evidence from Chinese college students
Source: Front Psychol. 2025 May 20;16:1501327. doi: 10.3389/fpsyg.2025.1501327 (PMC12129995; doi:10.3389/fpsyg.2025.1501327)
Supplement: Supplementary file 1 [file Data_Sheet_1.zip › Raw Data/path analysis.AmosOutput]

path analysis.amw


# \\Mac\Home\Desktop\Raw data\path analysis.amw

## Analysis Summary

## Date and Time

Date: 2024年9月28日

Time: 16:28:48

## Title

path analysis: 2024年9月28日 16:28

## Groups

## Group number 1 (Group number 1)

## Notes for Group (Group number 1)

The model is recursive.

Sample size = 861

## Variable Summary (Group number 1)

## Your model contains the following variables (Group number 1)

Observed, endogenous variables

DTU1

DTU2

DTU3

EE1

EE2

EE3

TSC1

TSC2

TSC3

PSC1

PSC2

PSC3

PSC4

SSC1

SSC2

Unobserved, endogenous variables

EE

TSC

PSC

SSC

Unobserved, exogenous variables

DTU

e1

e2

e3

e4

e5

e6

e7

e8

e9

e13

e14

e15

e16

e17

e18

e19

e20

e21

e22

## Variable counts (Group number 1)

|  |  |
| --- | --- |
| Number of variables in your model: | 39 |
| Number of observed variables: | 15 |
| Number of unobserved variables: | 24 |
| Number of exogenous variables: | 20 |
| Number of endogenous variables: | 19 |

## Parameter Summary (Group number 1)

|  | Weights | Covariances | Variances | Means | Intercepts | Total |
| --- | --- | --- | --- | --- | --- | --- |
| Fixed | 24 | 0 | 0 | 0 | 0 | 24 |
| Labeled | 0 | 0 | 0 | 0 | 0 | 0 |
| Unlabeled | 17 | 0 | 20 | 0 | 0 | 37 |
| Total | 41 | 0 | 20 | 0 | 0 | 61 |

## Models

## Default model (Default model)

## Notes for Model (Default model)

## Computation of degrees of freedom (Default model)

|  |  |
| --- | --- |
| Number of distinct sample moments: | 120 |
| Number of distinct parameters to be estimated: | 37 |
| Degrees of freedom (120 - 37): | 83 |

## Result (Default model)

Minimum was achieved

Chi-square = 252.078

Degrees of freedom = 83

Probability level = .000

## Group number 1 (Group number 1 - Default model)

## Estimates (Group number 1 - Default model)

## Scalar Estimates (Group number 1 - Default model)

## Maximum Likelihood Estimates

## Regression Weights: (Group number 1 - Default model)

|  |  |  | Estimate | S.E. | C.R. | P | Label |
| --- | --- | --- | --- | --- | --- | --- | --- |
| EE | <--- | DTU | .614 | .048 | 12.893 | \*\*\* | par\_11 |
| SSC | <--- | DTU | .382 | .053 | 7.165 | \*\*\* | par\_12 |
| TSC | <--- | DTU | .398 | .050 | 7.970 | \*\*\* | par\_13 |
| PSC | <--- | DTU | .381 | .047 | 8.036 | \*\*\* | par\_14 |
| TSC | <--- | EE | .324 | .047 | 6.902 | \*\*\* | par\_15 |
| PSC | <--- | EE | .252 | .044 | 5.760 | \*\*\* | par\_16 |
| SSC | <--- | EE | .259 | .050 | 5.221 | \*\*\* | par\_17 |
| DTU1 | <--- | DTU | 1.000 |  |
| DTU2 | <--- | DTU | .993 | .048 | 20.876 | \*\*\* | par\_1 |
| DTU3 | <--- | DTU | 1.008 | .052 | 19.285 | \*\*\* | par\_2 |
| EE1 | <--- | EE | 1.000 |  |
| EE2 | <--- | EE | 1.091 | .052 | 20.867 | \*\*\* | par\_3 |
| EE3 | <--- | EE | .872 | .046 | 19.076 | \*\*\* | par\_4 |
| TSC1 | <--- | TSC | 1.000 |  |
| TSC2 | <--- | TSC | .928 | .041 | 22.811 | \*\*\* | par\_5 |
| TSC3 | <--- | TSC | 1.071 | .046 | 23.447 | \*\*\* | par\_6 |
| PSC1 | <--- | PSC | 1.000 |  |
| PSC2 | <--- | PSC | 1.002 | .044 | 22.725 | \*\*\* | par\_7 |
| PSC3 | <--- | PSC | 1.003 | .044 | 22.954 | \*\*\* | par\_8 |
| PSC4 | <--- | PSC | 1.095 | .048 | 22.771 | \*\*\* | par\_9 |
| SSC1 | <--- | SSC | 1.000 |  |
| SSC2 | <--- | SSC | .933 | .061 | 15.224 | \*\*\* | par\_10 |

## Standardized Regression Weights: (Group number 1 - Default model)

|  |  |  | Estimate |
| --- | --- | --- | --- |
| EE | <--- | DTU | .584 |
| SSC | <--- | DTU | .383 |
| TSC | <--- | DTU | .392 |
| PSC | <--- | DTU | .402 |
| TSC | <--- | EE | .335 |
| PSC | <--- | EE | .280 |
| SSC | <--- | EE | .273 |
| DTU1 | <--- | DTU | .740 |
| DTU2 | <--- | DTU | .809 |
| DTU3 | <--- | DTU | .726 |
| EE1 | <--- | EE | .760 |
| EE2 | <--- | EE | .804 |
| EE3 | <--- | EE | .710 |
| TSC1 | <--- | TSC | .812 |
| TSC2 | <--- | TSC | .778 |
| TSC3 | <--- | TSC | .804 |
| PSC1 | <--- | PSC | .780 |
| PSC2 | <--- | PSC | .776 |
| PSC3 | <--- | PSC | .784 |
| PSC4 | <--- | PSC | .778 |
| SSC1 | <--- | SSC | .817 |
| SSC2 | <--- | SSC | .784 |

## Variances: (Group number 1 - Default model)

|  |  |  | Estimate | S.E. | C.R. | P | Label |
| --- | --- | --- | --- | --- | --- | --- | --- |
| DTU |  |  | .655 | .056 | 11.655 | \*\*\* | par\_18 |
| e19 |  |  | .476 | .044 | 10.787 | \*\*\* | par\_19 |
| e20 |  |  | .391 | .033 | 11.851 | \*\*\* | par\_20 |
| e21 |  |  | .370 | .031 | 11.879 | \*\*\* | par\_21 |
| e22 |  |  | .429 | .043 | 10.000 | \*\*\* | par\_22 |
| e1 |  |  | .542 | .035 | 15.714 | \*\*\* | par\_23 |
| e2 |  |  | .341 | .026 | 12.870 | \*\*\* | par\_24 |
| e3 |  |  | .597 | .037 | 16.119 | \*\*\* | par\_25 |
| e4 |  |  | .527 | .036 | 14.672 | \*\*\* | par\_26 |
| e5 |  |  | .472 | .037 | 12.739 | \*\*\* | par\_27 |
| e6 |  |  | .542 | .033 | 16.328 | \*\*\* | par\_28 |
| e7 |  |  | .348 | .026 | 13.419 | \*\*\* | par\_29 |
| e8 |  |  | .379 | .025 | 14.997 | \*\*\* | par\_30 |
| e9 |  |  | .422 | .031 | 13.810 | \*\*\* | par\_31 |
| e13 |  |  | .379 | .024 | 15.753 | \*\*\* | par\_32 |
| e14 |  |  | .389 | .025 | 15.862 | \*\*\* | par\_33 |
| e15 |  |  | .371 | .024 | 15.609 | \*\*\* | par\_34 |
| e16 |  |  | .460 | .029 | 15.812 | \*\*\* | par\_35 |
| e17 |  |  | .325 | .041 | 7.925 | \*\*\* | par\_36 |
| e18 |  |  | .356 | .037 | 9.566 | \*\*\* | par\_37 |

## Squared Multiple Correlations: (Group number 1 - Default model)

|  |  |  | Estimate |
| --- | --- | --- | --- |
| EE |  |  | .341 |
| SSC |  |  | .343 |
| PSC |  |  | .371 |
| TSC |  |  | .420 |
| SSC2 |  |  | .615 |
| SSC1 |  |  | .668 |
| PSC4 |  |  | .605 |
| PSC3 |  |  | .615 |
| PSC2 |  |  | .603 |
| PSC1 |  |  | .608 |
| TSC3 |  |  | .647 |
| TSC2 |  |  | .605 |
| TSC1 |  |  | .659 |
| EE3 |  |  | .503 |
| EE2 |  |  | .646 |
| EE1 |  |  | .578 |
| DTU3 |  |  | .527 |
| DTU2 |  |  | .655 |
| DTU1 |  |  | .547 |

## Modification Indices (Group number 1 - Default model)

## Covariances: (Group number 1 - Default model)

|  |  |  | M.I. | Par Change |
| --- | --- | --- | --- | --- |
| e21 | <--> | e22 | 33.063 | .109 |
| e20 | <--> | e22 | 18.457 | .087 |
| e20 | <--> | e21 | 48.225 | .123 |
| e18 | <--> | e21 | 8.025 | .049 |
| e18 | <--> | e20 | 5.662 | .043 |
| e17 | <--> | e21 | 6.822 | .046 |
| e16 | <--> | DTU | 9.103 | .069 |
| e16 | <--> | e22 | 5.141 | .047 |
| e16 | <--> | e21 | 5.771 | -.042 |
| e16 | <--> | e20 | 24.240 | .094 |
| e15 | <--> | e16 | 11.886 | -.059 |
| e14 | <--> | e20 | 6.712 | .046 |
| e13 | <--> | e15 | 10.839 | .051 |
| e9 | <--> | e22 | 7.167 | .055 |
| e9 | <--> | e21 | 16.365 | .073 |
| e9 | <--> | e18 | 9.604 | .058 |
| e9 | <--> | e16 | 18.929 | .085 |
| e9 | <--> | e13 | 5.032 | -.040 |
| e8 | <--> | e22 | 6.049 | .047 |
| e8 | <--> | e13 | 5.551 | .039 |
| e7 | <--> | e21 | 5.991 | .041 |
| e7 | <--> | e14 | 5.603 | .039 |
| e6 | <--> | e14 | 5.551 | -.045 |
| e6 | <--> | e9 | 9.915 | -.066 |
| e4 | <--> | e15 | 5.928 | -.047 |
| e3 | <--> | e20 | 4.105 | -.044 |
| e3 | <--> | e8 | 4.514 | -.043 |
| e1 | <--> | e21 | 9.495 | -.060 |
| e1 | <--> | e13 | 7.735 | -.054 |

## Variances: (Group number 1 - Default model)

|  |  |  | M.I. | Par Change |
| --- | --- | --- | --- | --- |

## Regression Weights: (Group number 1 - Default model)

|  |  |  | M.I. | Par Change |
| --- | --- | --- | --- | --- |
| SSC | <--- | PSC | 18.058 | .162 |
| SSC | <--- | TSC | 8.902 | .107 |
| PSC | <--- | SSC | 18.322 | .141 |
| PSC | <--- | TSC | 23.277 | .152 |
| TSC | <--- | SSC | 10.231 | .112 |
| TSC | <--- | PSC | 26.368 | .182 |
| SSC2 | <--- | PSC | 4.369 | .072 |
| SSC2 | <--- | PSC2 | 4.484 | .053 |
| SSC2 | <--- | TSC3 | 7.919 | .063 |
| SSC1 | <--- | PSC4 | 5.198 | .053 |
| PSC4 | <--- | DTU | 9.103 | .105 |
| PSC4 | <--- | EE | 5.344 | .077 |
| PSC4 | <--- | SSC | 12.332 | .125 |
| PSC4 | <--- | TSC | 28.670 | .182 |
| PSC4 | <--- | SSC2 | 7.557 | .074 |
| PSC4 | <--- | SSC1 | 10.684 | .085 |
| PSC4 | <--- | TSC3 | 38.397 | .147 |
| PSC4 | <--- | TSC2 | 10.801 | .087 |
| PSC4 | <--- | TSC1 | 21.631 | .119 |
| PSC4 | <--- | DTU3 | 7.576 | .063 |
| PSC4 | <--- | DTU1 | 6.228 | .059 |
| PSC3 | <--- | PSC4 | 4.003 | -.043 |
| PSC2 | <--- | TSC1 | 5.164 | .053 |
| PSC2 | <--- | EE3 | 4.576 | -.049 |
| PSC1 | <--- | TSC3 | 4.920 | -.048 |
| PSC1 | <--- | DTU1 | 8.936 | -.064 |
| TSC3 | <--- | SSC | 4.573 | .076 |
| TSC3 | <--- | PSC | 9.863 | .114 |
| TSC3 | <--- | SSC2 | 9.553 | .083 |
| TSC3 | <--- | PSC4 | 22.322 | .113 |
| TSC3 | <--- | PSC3 | 9.554 | .082 |
| TSC3 | <--- | PSC2 | 6.376 | .066 |
| TSC3 | <--- | EE3 | 6.420 | -.063 |
| TSC1 | <--- | PSC4 | 4.537 | .047 |
| TSC1 | <--- | PSC2 | 7.545 | .066 |
| EE3 | <--- | PSC2 | 6.722 | -.072 |
| EE3 | <--- | TSC3 | 6.965 | -.066 |
| DTU3 | <--- | TSC2 | 4.150 | -.060 |
| DTU1 | <--- | PSC | 5.443 | -.091 |
| DTU1 | <--- | PSC2 | 4.731 | -.061 |
| DTU1 | <--- | PSC1 | 10.583 | -.092 |

## Minimization History (Default model)

| Iteration |  | Negative eigenvalues | Condition # | Smallest eigenvalue | Diameter | F | NTries | Ratio |
| --- | --- | --- | --- | --- | --- | --- | --- | --- |
| 0 | e | 10 |  | -.476 | 9999.000 | 6130.512 | 0 | 9999.000 |
| 1 | e\* | 5 |  | -.113 | 3.031 | 2366.675 | 20 | .434 |
| 2 | e | 0 | 257.359 |  | 1.445 | 691.791 | 4 | .745 |
| 3 | e | 0 | 68.526 |  | .585 | 491.523 | 4 | .000 |
| 4 | e | 0 | 156.387 |  | .966 | 456.828 | 1 | .140 |
| 5 | e | 0 | 41.353 |  | .317 | 281.549 | 1 | 1.111 |
| 6 | e | 0 | 23.748 |  | .267 | 254.968 | 1 | 1.115 |
| 7 | e | 0 | 22.604 |  | .059 | 252.127 | 1 | 1.072 |
| 8 | e | 0 | 22.181 |  | .011 | 252.078 | 1 | 1.013 |
| 9 | e | 0 | 22.343 |  | .000 | 252.078 | 1 | 1.000 |

## Pairwise Parameter Comparisons (Default model)

## Variance-covariance Matrix of Estimates (Default model)

|  | par\_1 | par\_2 | par\_3 | par\_4 | par\_5 | par\_6 | par\_7 | par\_8 | par\_9 | par\_10 | par\_11 | par\_12 | par\_13 | par\_14 | par\_15 | par\_16 | par\_17 | par\_18 | par\_19 | par\_20 | par\_21 | par\_22 | par\_23 | par\_24 | par\_25 | par\_26 | par\_27 | par\_28 | par\_29 | par\_30 | par\_31 | par\_32 | par\_33 | par\_34 | par\_35 | par\_36 | par\_37 |
| --- | --- | --- | --- | --- | --- | --- | --- | --- | --- | --- | --- | --- | --- | --- | --- | --- | --- | --- | --- | --- | --- | --- | --- | --- | --- | --- | --- | --- | --- | --- | --- | --- | --- | --- | --- | --- | --- |
| par\_1 | .002 |
| par\_2 | .001 | .003 |
| par\_3 | .000 | .000 | .003 |
| par\_4 | .000 | .000 | .001 | .002 |
| par\_5 | .000 | .000 | .000 | .000 | .002 |
| par\_6 | .000 | .000 | .000 | .000 | .001 | .002 |
| par\_7 | .000 | .000 | .000 | .000 | .000 | .000 | .002 |
| par\_8 | .000 | .000 | .000 | .000 | .000 | .000 | .001 | .002 |
| par\_9 | .000 | .000 | .000 | .000 | .000 | .000 | .001 | .001 | .002 |
| par\_10 | .000 | .000 | .000 | .000 | .000 | .000 | .000 | .000 | .000 | .004 |
| par\_11 | .001 | .001 | -.001 | -.001 | .000 | .000 | .000 | .000 | .000 | .000 | .002 |
| par\_12 | .000 | .001 | .000 | .000 | .000 | .000 | .000 | .000 | .000 | -.001 | .000 | .003 |
| par\_13 | .001 | .001 | .000 | .000 | .000 | .000 | .000 | .000 | .000 | .000 | .000 | .000 | .002 |
| par\_14 | .000 | .001 | .000 | .000 | .000 | .000 | .000 | .000 | .000 | .000 | .000 | .000 | .000 | .002 |
| par\_15 | .000 | .000 | .000 | .000 | .000 | .000 | .000 | .000 | .000 | .000 | .000 | .000 | -.001 | .000 | .002 |
| par\_16 | .000 | .000 | .000 | .000 | .000 | .000 | .000 | .000 | .000 | .000 | .000 | .000 | .000 | -.001 | .000 | .002 |
| par\_17 | .000 | .000 | .000 | .000 | .000 | .000 | .000 | .000 | .000 | .000 | .000 | -.002 | .000 | .000 | .000 | .000 | .002 |
| par\_18 | -.002 | -.002 | .000 | .000 | .000 | .000 | .000 | .000 | .000 | .000 | -.001 | -.001 | -.001 | -.001 | .000 | .000 | .000 | .003 |
| par\_19 | .000 | .000 | -.001 | -.001 | .000 | .000 | .000 | .000 | .000 | .000 | .000 | .000 | .000 | .000 | .000 | .000 | .000 | .000 | .002 |
| par\_20 | .000 | .000 | .000 | .000 | -.001 | -.001 | .000 | .000 | .000 | .000 | .000 | .000 | .000 | .000 | .000 | .000 | .000 | .000 | .000 | .001 |
| par\_21 | .000 | .000 | .000 | .000 | .000 | .000 | -.001 | -.001 | -.001 | .000 | .000 | .000 | .000 | .000 | .000 | .000 | .000 | .000 | .000 | .000 | .001 |
| par\_22 | .000 | .000 | .000 | .000 | .000 | .000 | .000 | .000 | .000 | -.002 | .000 | .000 | .000 | .000 | .000 | .000 | .000 | .000 | .000 | .000 | .000 | .002 |
| par\_23 | .000 | .000 | .000 | .000 | .000 | .000 | .000 | .000 | .000 | .000 | .000 | .000 | .000 | .000 | .000 | .000 | .000 | -.001 | .000 | .000 | .000 | .000 | .001 |
| par\_24 | .000 | .000 | .000 | .000 | .000 | .000 | .000 | .000 | .000 | .000 | .000 | .000 | .000 | .000 | .000 | .000 | .000 | .000 | .000 | .000 | .000 | .000 | .000 | .001 |
| par\_25 | .000 | .000 | .000 | .000 | .000 | .000 | .000 | .000 | .000 | .000 | .000 | .000 | .000 | .000 | .000 | .000 | .000 | .000 | .000 | .000 | .000 | .000 | .000 | .000 | .001 |
| par\_26 | .000 | .000 | .001 | .000 | .000 | .000 | .000 | .000 | .000 | .000 | .000 | .000 | .000 | .000 | .000 | .000 | .000 | .000 | .000 | .000 | .000 | .000 | .000 | .000 | .000 | .001 |
| par\_27 | .000 | .000 | -.001 | .000 | .000 | .000 | .000 | .000 | .000 | .000 | .000 | .000 | .000 | .000 | .000 | .000 | .000 | .000 | .000 | .000 | .000 | .000 | .000 | .000 | .000 | .000 | .001 |
| par\_28 | .000 | .000 | .000 | .000 | .000 | .000 | .000 | .000 | .000 | .000 | .000 | .000 | .000 | .000 | .000 | .000 | .000 | .000 | .000 | .000 | .000 | .000 | .000 | .000 | .000 | .000 | .000 | .001 |
| par\_29 | .000 | .000 | .000 | .000 | .000 | .000 | .000 | .000 | .000 | .000 | .000 | .000 | .000 | .000 | .000 | .000 | .000 | .000 | .000 | .000 | .000 | .000 | .000 | .000 | .000 | .000 | .000 | .000 | .001 |
| par\_30 | .000 | .000 | .000 | .000 | .000 | .000 | .000 | .000 | .000 | .000 | .000 | .000 | .000 | .000 | .000 | .000 | .000 | .000 | .000 | .000 | .000 | .000 | .000 | .000 | .000 | .000 | .000 | .000 | .000 | .001 |
| par\_31 | .000 | .000 | .000 | .000 | .000 | .000 | .000 | .000 | .000 | .000 | .000 | .000 | .000 | .000 | .000 | .000 | .000 | .000 | .000 | .000 | .000 | .000 | .000 | .000 | .000 | .000 | .000 | .000 | .000 | .000 | .001 |
| par\_32 | .000 | .000 | .000 | .000 | .000 | .000 | .000 | .000 | .000 | .000 | .000 | .000 | .000 | .000 | .000 | .000 | .000 | .000 | .000 | .000 | .000 | .000 | .000 | .000 | .000 | .000 | .000 | .000 | .000 | .000 | .000 | .001 |
| par\_33 | .000 | .000 | .000 | .000 | .000 | .000 | .000 | .000 | .000 | .000 | .000 | .000 | .000 | .000 | .000 | .000 | .000 | .000 | .000 | .000 | .000 | .000 | .000 | .000 | .000 | .000 | .000 | .000 | .000 | .000 | .000 | .000 | .001 |
| par\_34 | .000 | .000 | .000 | .000 | .000 | .000 | .000 | .000 | .000 | .000 | .000 | .000 | .000 | .000 | .000 | .000 | .000 | .000 | .000 | .000 | .000 | .000 | .000 | .000 | .000 | .000 | .000 | .000 | .000 | .000 | .000 | .000 | .000 | .001 |
| par\_35 | .000 | .000 | .000 | .000 | .000 | .000 | .000 | .000 | .000 | .000 | .000 | .000 | .000 | .000 | .000 | .000 | .000 | .000 | .000 | .000 | .000 | .000 | .000 | .000 | .000 | .000 | .000 | .000 | .000 | .000 | .000 | .000 | .000 | .000 | .001 |
| par\_36 | .000 | .000 | .000 | .000 | .000 | .000 | .000 | .000 | .000 | .002 | .000 | .000 | .000 | .000 | .000 | .000 | .000 | .000 | .000 | .000 | .000 | -.001 | .000 | .000 | .000 | .000 | .000 | .000 | .000 | .000 | .000 | .000 | .000 | .000 | .000 | .002 |
| par\_37 | .000 | .000 | .000 | .000 | .000 | .000 | .000 | .000 | .000 | -.002 | .000 | .000 | .000 | .000 | .000 | .000 | .000 | .000 | .000 | .000 | .000 | .001 | .000 | .000 | .000 | .000 | .000 | .000 | .000 | .000 | .000 | .000 | .000 | .000 | .000 | -.001 | .001 |

## Model Fit Summary

## CMIN

| Model | NPAR | CMIN | DF | P | CMIN/DF |
| --- | --- | --- | --- | --- | --- |
| Default model | 37 | 252.078 | 83 | .000 | 3.037 |
| Saturated model | 120 | .000 | 0 |
| Independence model | 15 | 5948.419 | 105 | .000 | 56.652 |

## RMR, GFI

| Model | RMR | GFI | AGFI | PGFI |
| --- | --- | --- | --- | --- |
| Default model | .058 | .962 | .945 | .665 |
| Saturated model | .000 | 1.000 |  |  |
| Independence model | .386 | .331 | .236 | .290 |

## Baseline Comparisons

| Model | NFI Delta1 | RFI rho1 | IFI Delta2 | TLI rho2 | CFI |
| --- | --- | --- | --- | --- | --- |
| Default model | .958 | .946 | .971 | .963 | .971 |
| Saturated model | 1.000 |  | 1.000 |  | 1.000 |
| Independence model | .000 | .000 | .000 | .000 | .000 |

## Parsimony-Adjusted Measures

| Model | PRATIO | PNFI | PCFI |
| --- | --- | --- | --- |
| Default model | .790 | .757 | .768 |
| Saturated model | .000 | .000 | .000 |
| Independence model | 1.000 | .000 | .000 |

## NCP

| Model | NCP | LO 90 | HI 90 |
| --- | --- | --- | --- |
| Default model | 169.078 | 125.102 | 220.682 |
| Saturated model | .000 | .000 | .000 |
| Independence model | 5843.419 | 5593.988 | 6099.165 |

## FMIN

| Model | FMIN | F0 | LO 90 | HI 90 |
| --- | --- | --- | --- | --- |
| Default model | .293 | .197 | .145 | .257 |
| Saturated model | .000 | .000 | .000 | .000 |
| Independence model | 6.917 | 6.795 | 6.505 | 7.092 |

## RMSEA

| Model | RMSEA | LO 90 | HI 90 | PCLOSE |
| --- | --- | --- | --- | --- |
| Default model | .049 | .042 | .056 | .613 |
| Independence model | .254 | .249 | .260 | .000 |

## AIC

| Model | AIC | BCC | BIC | CAIC |
| --- | --- | --- | --- | --- |
| Default model | 326.078 | 327.480 | 502.127 | 539.127 |
| Saturated model | 240.000 | 244.550 | 810.971 | 930.971 |
| Independence model | 5978.419 | 5978.988 | 6049.791 | 6064.791 |

## ECVI

| Model | ECVI | LO 90 | HI 90 | MECVI |
| --- | --- | --- | --- | --- |
| Default model | .379 | .328 | .439 | .381 |
| Saturated model | .279 | .279 | .279 | .284 |
| Independence model | 6.952 | 6.662 | 7.249 | 6.952 |

## HOELTER

| Model | HOELTER .05 | HOELTER .01 |
| --- | --- | --- |
| Default model | 360 | 396 |
| Independence model | 19 | 21 |

## Execution time summary

|  |  |
| --- | --- |
| Minimization: | .174 |
| Miscellaneous: | .830 |
| Bootstrap: | .000 |
| Total: | 1.004 |
